# The clonal repopulation of HSPC gene modified with anti–HIV-1 RNAi is not affected by preexisting HIV-1 infection

**DOI:** 10.1126/sciadv.aay9206

**Published:** 2020-07-22

**Authors:** Gajendra W. Suryawanshi, Wannisa Khamaikawin, Jing Wen, Saki Shimizu, Hubert Arokium, Yiming Xie, Eugene Wang, Shihyoung Kim, Hyewon Choi, Chong Zhang, Hannah Yu, Angela P. Presson, Namshin Kim, Dong-Sung An, Irvin S. Y. Chen, Sanggu Kim

**Affiliations:** 1 Department of Microbiology, Immunology and Molecular Genetics, University of California, Los Angeles, Los Angeles, CA 90095, USA.; 2 UCLA AIDS Institute, Los Angeles, CA 90095, USA.; 3 School of Nursing, University of California, Los Angeles, CA 90095, USA.; 4 Department of Veterinary Biosciences, College of Veterinary Medicine, The Ohio State University, Columbus, OH 43210, USA.; 5 Center for Retrovirus Research, The Ohio State University, Columbus, OH 43210, USA.; 6 Infectious Disease Institute, The Ohio State University, Columbus, OH 43210, USA.; 7 Division of Epidemiology, Department of Internal Medicine, University of Utah, Salt Lake City, UT 84108, USA.; 8 Department of Biostatistics, University of California, Los Angeles, CA 90095, USA.; 9 Genome Editing Research Center, Korea Research Institute of Biosciences and Biotechnology, Daejeon 34141, Republic of Korea.; 10 Division of Hematology-Oncology, David Geffen School of Medicine, UCLA, Los Angeles, CA 90095, USA.

## Abstract

Despite advances in hematopoietic stem/progenitor cell (HSPC) transplant for HIV-1–infected patients, the impact of a preexisting HIV-1 infection on the engraftment and clonal repopulation of HSPCs remains poorly understood. We have developed a long terminal repeat indexing-mediated integration site sequencing (LTRi-Seq) method that provides a multiplexed clonal quantitation of both anti–HIV-1 RNAi (RNA interference) gene-modified and control vector-modified cell populations, together with HIV-1–infected cells—all within the same animal. In our HIV-1–preinfected humanized mice, both therapeutic and control HSPCs repopulated efficiently without abnormalities. Although the HIV-1–mediated selection of anti–HIV-1 RNAi-modified clones was evident in HIV-1–infected mice, the organ-to-organ and intra-organ clonal distributions in infected mice were indistinguishable from those in uninfected mice. HIV-1–infected cells showed clonal patterns distinct from those of HSPCs. Our data demonstrate that, despite the substantial impact of HIV-1 infection on CD4^+^ T cells, HSPC repopulation remains polyclonal, thus supporting the use of HSPC transplant for anti-HIV treatment.

## Teaser

A novel approach enables a direct comparison of anti-HIV– and non–gene-modified stem-cell engraftment in an HIV-1–infected host.

## INTRODUCTION

The widespread availability of combination antiretroviral therapy (cART) has significantly reduced AIDS-related mortality and morbidity. Thanks to improved cART and patient care, high-dose therapy and hematopoietic stem/progenitor cell (HSPC) transplantation, once considered too risky for HIV-infected patients, are now increasingly used in clinic to treat malignancies in patients with HIV (*1*–*6*). A number of recent studies have demonstrated a level of clinical efficacy for HSPC transplant in patients with HIV that is similar to the efficacy in noninfected patients (*4*, *7*). Furthermore, two remarkable case studies—the so-called “Berlin patient,” the first case cured of HIV after the allogeneic transplantation of HIV-resistant (*CCR5*∆*32*/∆*32*) bone marrow (BM) (*8*), and the recent “London patient,” potentially the second cure with the same transplant strategy (*9*)—have generated tremendous hope that HIV can be treated by the genetic engineering of a patient’s own HSPC (*10*–*13*). Despite these recent clinical successes, however, our understanding of the functions of transplanted HSPC in HIV-infected patients remains unclear and controversial. In particular, a short-term cART interruption, recently recommended to minimize transplant-associated problems, often results in a marked increase in viral load in patients (*2*, *3*, *14*), but the impact of ongoing viral replication on HSPC engraftment and tissue repopulation remains poorly understood. It is noteworthy that numerous previous reports have shown both direct and indirect effects of HIV infection on BM niche cells, including HSPC (*15*), stromal cells (*16*), and possibly, CD4^+^ T cells (*17*). Furthermore, recent nonhuman primate studies have identified perturbations in the immune system following HSPC transplant in simian HIV (SHIV)–infected, ART-suppressed animals (*14*, *18*, *19*). Nevertheless, most in vivo preclinical studies so far have tested HSPC transplant in the absence of HIV-1 infection (*20*–*26*). All current and planned trials that include a cART interruption are moving forward without a full understanding of the effects of HIV infection on HSPC behaviors in vivo.

Retroviral tagging (cellular barcoding) has proven useful in evaluating HSPC transplant and the effects of genetic modification on HSPC behaviors in vivo (*27*). Previous human and nonhuman primate studies using the traditional reporter gene and polymerase chain reaction (PCR) assays have provided only limited information on HSPC behaviors in the presence of HIV-1 (*2*, *3*, *5*) or simian virus infection (*14*, *18*, *19*, *25*), as these assays measure gene-marked cells as a whole population and thus overlook the clonal complexity that exists within the cells. In contrast, recent retroviral tagging studies, including our own, have shown highly coordinated repopulation by hundreds or thousands of individual repopulating HSPC clones at the systems level (*28*–*32*). Quantitative sequencing of vector integration sites (ISs), in particular, has been an excellent means of studying the safety and functional diversities of gene-modified CD34^+^ HSPCs in gene therapy settings (*30*–*36*). Although the importance of the tremendous regenerative potential and functional heterogeneity of individual hematopoietic stem cells has been well recognized (*32*, *37*), all previous studies have tested hematopoietic reconstitution either in the absence of HIV infection or in the presence of suppressive ART. Thus, polyclonal HSPC repopulation, an important indicator of normal HSPC homing and in vivo function, in the presence of HIV-1 infection remains uncharacterized.

A humanized BM/liver/thymus (BLT) mouse model is arguably the most practical and functional small-animal model with which to test HSPC transplant (*38*). These mice enable human HSPCs to proliferate and populate the BM, generate various mature and functioning immune lineages, including mature, functional T cells through the transplanted thymic tissue, and repopulate all the lymphoid and nonlymphoid organs (*20*). We have previously demonstrated effective tissue repopulation and the anti-HIV efficacy of gene-modified HSPCs in BLT mice (*21*–*23*) using several different types of anti-HIV lentiviral vectors, including dual-combination anti-HIV lentiviral vectors (“dual-sh1005/sh516”) expressing two anti-HIV short hairpin RNAs (shRNA), one directed at the HIV coreceptor CCR5 (sh1005) and the other at the viral long terminal repeat (LTR) (sh516). These dual-combination vectors showed antiviral efficacy against both R5- and X4-tropic HIV-1 in vivo (*21*). More recently, we developed a new “preinfection” BLT mouse model with which the HSPC transplant can be tested in the presence of HIV-1 infection (*39*). Gene-marking analysis in these mice has revealed the selective advantage of dual-sh1005/sh516–engineered T cells over control (nonprotective) HSPCs cotransplanted in the same animal. However, it remains unclear whether and how HIV-1 infection affects polyclonal engraftment of nonprotective HSPC and whether selective repopulation by anti–HIV gene–modified HSPC occurs via normal polyclonal hematopoiesis.

Here, we have developed a novel LTR indexing–mediated IS sequencing (LTRi-Seq) to directly compare and evaluate the homing/engraftment and tissue repopulation of anti-HIV (dual-sh1005/sh516) and control (nonprotective) HSPC in HIV-1–preinfected humanized mice. The LTRi-Seq enables unbiased, simultaneous analysis of both anti-HIV and control HSPC clones and HIV-1–infected cell clones in the same sample. With the new assay, our study provides novel insights into the competitive repopulation of HSPC clones in HIV-1-infected (HIV+) mice and the cellular proliferation and circulation of HIV-1–infected cell clones in the same animals.

## RESULTS

### A novel HIV-1–preinfected humanized mouse model reveals a selective advantage of HIV-protected T cells over nonprotected ones

**Fig. 1:**
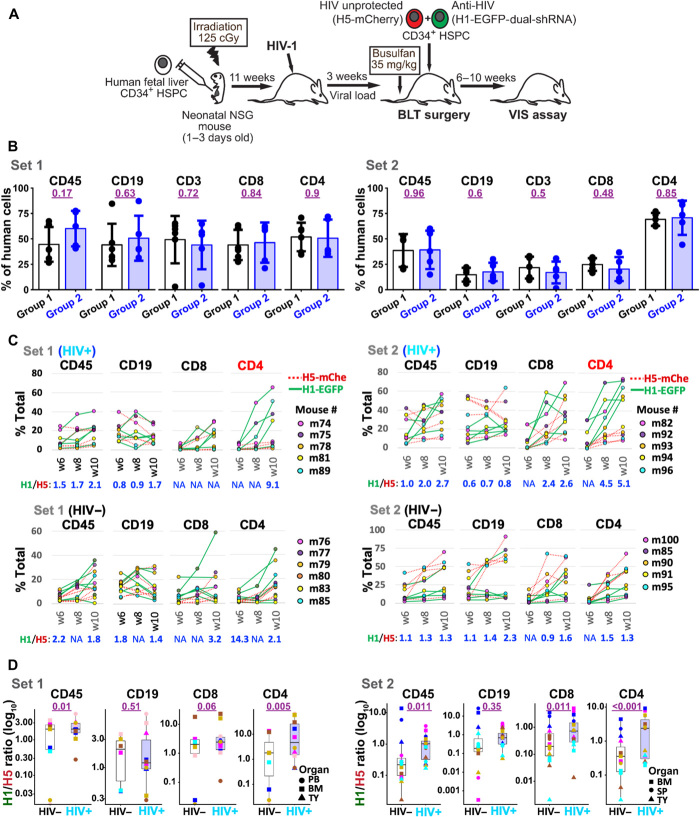
Selective expansion of HIV-1–protected CD4^+^ T cells in preinfection BLT mouse model. (**A**) Cartoon representation of HIV preinfected BLT mouse model. (**B**) Bar plots showing comparative levels of reconstitution of different human cell populations before HIV-1 infection (10 weeks after the first CD34^+^ cell transplant) in the two groups of humanized mice. Group 1 animals were used as an HIV− control and group 2 animals were infected with HIV-1 on week 11. Set 1 data are shown on the left and set 2 are on the right. Human cell reconstitution levels in groups 1 and 2 mice were similar; *P* values from Student’s *t* tests with Welch correction comparing groups 1 and 2 for human leukocytes (CD45), T cells (CD3), T helper cells (CD4), cytotoxic T cells (CD8), and B cells (CD19) are denoted in purple digits. (**C**) Gradual enrichment of anti-HIV (H1-EGFP-dual-shRNA)–modified T cells in the peripheral blood of HIV+ mice. The line charts show the percentages of EGFP^+^ (circles connected with a solid green line) and mCherry^+^ cells (circles connected with a dotted red line) in CD45, CD3, CD4, CD8, and CD19 cells at 6, 8, and 10 weeks (w6, w8, and w10) after BLT surgery. HIV-1–infected (HIV+, top) and mock-infected (HIV−, bottom) mice for sets 1 and 2 are shown separately. The colors of the circles match the mouse IDs. The average of paired EGFP-to-mCherry marking ratios (H1/H5) for all samples is shown at the bottom of each line chart. The average value is not available (NA) when there is any missing sample data. (**D**) Boxplots show H1-EGFP/H5-mCherry marking ratios (H1/H5) in each tissue sample, including BM (squares), thymic organelle (TY; triangles), peripheral blood (PB; circles in set 1), and spleen (SP; circles in set 2), at the end point (12 weeks after BLT surgery). The *P* values in purple were calculated by comparing the H1/H5 ratios between HIV+ and HIV− mice using mixed-effects gamma regressions (fig. S8A).

Two sets of independent preinfection humanized mouse experiments were performed at different times to evaluate and validate the impact of HIV-1 infection on HSPC transplant (Fig. 1A). The first set included five HIV+ mice and six uninfected (HIV–) control mice; the second set included five infected and five HIV– control mice. The details of the procedures and experimental results are described elsewhere (*39*). Briefly, human fetal liver CD34^+^ HSPCs were injected into irradiated neonatal (1 to 3 days old) nonobese diabetic.Cg-*Prkdc^scid^Il2rg^tm1Wjl^*/SzJ (NSG) mice. At 11 weeks after the first HSPC transplant—by which point human CD45^+^ cells, including CD19 B cells and CD3^+^CD4^+^ and CD8^+^ T cells, had repopulated the blood—half of the mice were infected with CCR5-tropic HIV-1_NFNSX_, while the rest remained uninfected as a control (Fig. 1B). After 3 weeks of infection, the viral load reached 5.10 × 10^5^ copies/ml (±1.48 × 10^5^ SD) for set 1 and 2.39 × 10^7^ copies/ml (±1.45 × 10^7^ SD) for set 2 (table S1). Both groups of mice were then subjected to BLT surgery, in which an equal mixture of two pools of human HSPC—one engineered with anti-HIV vectors (H1-EGFP-dual-shRNA) and the other with control vectors (H5-mCherry)—and a piece of human thymus tissue, all from the same donor, were transplanted into each mouse following the administration of busulfan the previous day. The animals were followed for an additional 12 weeks and euthanized for tissue repopulation analysis. We and others have shown stable multilineage human cell engraftment and gene marking at 10 to 12 weeks following HSPC transplant in NSG and BLT mice (*21*, *40*–*42*).

While showing marked mouse-to-mouse variations in human cell repopulation and experiment-to-experiment variations in the baseline gene markings, both sets 1 and 2 experiments demonstrated a gradual enrichment of enhanced green fluorescent protein–positive (EGFP^+^) cells over time within the CD4^+^ T cell population in HIV+ mice (Fig. 1C). These experimental variations are common in BLT mouse studies likely due to donor variations and inherent technical inconsistency in performing the BLT surgery (*38*, *43*). To effectively evaluate the impact of HIV-1 infection in our BLT mouse experiments, we compared paired EGFP versus mCherry marking levels in each sample, taking advantage of our cotransplantation strategy that provides an internal control (mCherry^+^ cells) in each mouse, using mixed-effects gamma regression models (see Materials and Methods for statistical analysis and fig. S8 for more details). At 12 weeks after the BLT surgery, despite the differences in the baseline data, HIV+ mice in both sets 1 and 2 showed a significant selective advantage for anti–HIV gene–engineered (EGFP^+^) cells over control (mCherry^+^) cells within the CD4^+^ T cell population (Fig. 1D and fig. S1E), demonstrating 7.6-fold higher EGFP/mCherry ratios than those of HIV− mice for set 1 and 3.9-fold higher EGFP/mCherry ratios for set 2 (*P =* 0.005 for the first set; *P =* <0.001 for the second set). Other cell types, including human CD45^+^, CD19^+^ B cells, and CD3^+^CD8^+^ T cells, did not show such significant differences, indicating that the HIV-1_NFNSX_–mediated selection was largely limited to mature CD4^+^ T cells (Fig. 1, C and D, and fig. S1). We have previously shown that CCR5^+^CD4^+^ T cells were primarily depleted in HIV-1_NFNSX_–infected mice (*39*). Lentiviral expression of dual-shRNA (sh516 and sh1005) had no obvious cytotoxicity in our previous tests (*21*). Most of the infected mice showed a similar or slightly increased viral load at the end point compared with their viral load before BLT surgery (table S1), indicating that viral replication continued until the end point.

### LTR indexing enabled efficient, paralleled IS sequencing for anti-HIV and control lentiviral vectors and HIV-1 proviruses

**Fig. 2:**
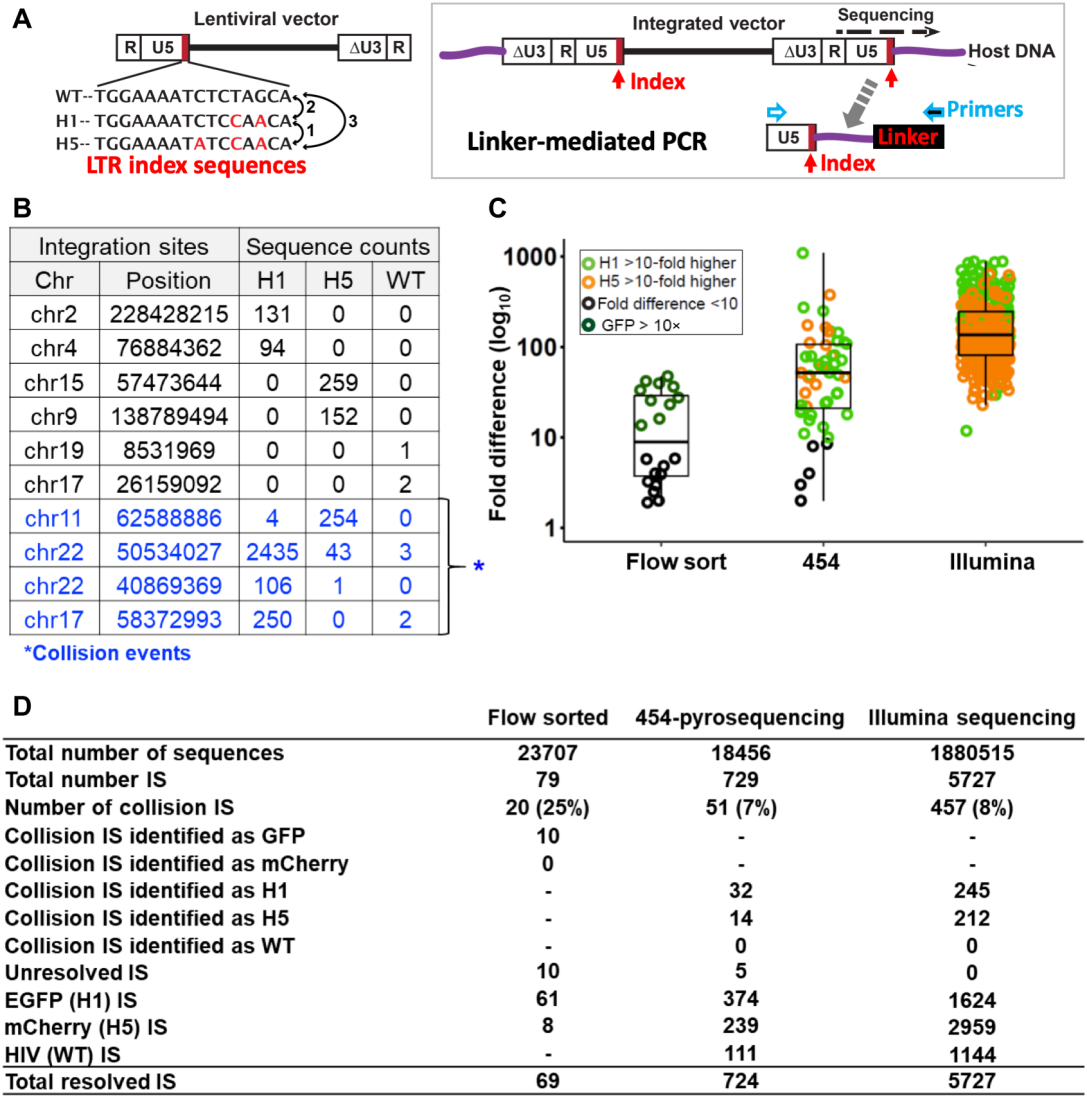
A multiplex LTR indexing method for parallel IS identification of H1 and H5 vectors and HIV-1. (**A**) A diagram showing LTR index sequences for the 3′-end U5 region (red box) of HIV, H1-EGFP-dual-shRNA, and H5-mCherry LTR. H1 and H5 indexes are two and three bases different, respectively, than the wild-type (WT) HIV-1. These unique index sequences will appear at the junction of the vector and host DNA after vector integration into the host genome and will thus serve as a marker with which to distinguish vector types during vector IS sequence analysis. The IS sequencing method is shown in the box. The LTR genome junction DNAs were PCR amplified by a linker-mediated PCR method using LTR- and linker-specific primers (blue-lined arrows) and then subjected to next-generation sequencing. (**B**) Table showing representative IS data with examples of collision events, a unique IS detected with multiple indexes (H1, H5, or WT). (**C**) Fold differences in IS sequence counts between the highest sequence count and the second highest sequence count for IS collision events detected in set 1 Roche 454-pyrosequencing and set 2 Illumina MiSeq sequencing data. For the sake of comparison, IS data from conventional IS sequencing (non–LTR indexed) using flow-sorted samples (flow sort) are shown. (**D**) A summary table for IS sequence analysis. Of the 729 unique ISs recovered in set 1 experiments and the 5727 unique ISs in set 2 experiments, approximately 7 and 8%, respectively, were collision events. Only five of the set 1 and none of the set 2 collision events showed a less than 10-fold difference; these remained unresolved even after we had conducted a selection process to determine the true index of the collision events.

The clonal-level evaluation of HSPC transplant, unachievable with conventional gene-marking or PCR assays, has been enabled by vector IS sequence analysis. The analysis of IS of multiple types of vectors in the same animal, however, remains challenging. To analyze the competitive clonal repopulation of anti-HIV and control HSPCs, we developed LTRi-Seq using two index sequences (H1 and H5) uniquely labeled at the U5 end of the LTR of the two lentivectors (see Fig. 2A). Distinguishing the unique LTR index sequences for these two vectors and the wild-type LTR of HIV-1 proviruses during IS sequence analysis enabled multiplexed and unbiased analysis of these two vectors and HIV-1 proviruses, all in parallel. The index sequences (H1 and H5) in the LTR did not induce a significant reduction in the efficiency of vector production or vector infectivity, nor did these indexes alter the genomic IS patterns of the lentiviral vectors (fig. S2).

In an effort to minimize the technical biases that can arise when comparing sample-to-sample IS profiles, we used the same amount of genomic DNA for all of the tissue samples (1 μg for set 1 and 2 μg for set 2 samples), with only a few exceptions (see table S2), and followed a well-established standard operating procedure for IS sequencing and quantitation (*32*, *44*). Sets 1 and 2 samples were sequenced using different sequencing platforms, 454-pyrosequencing for set 1 and MiSeq sequencing for set 2, with the IS amplification and sequencing procedures kept identical for each set. A total of 729 and 5727 unique ISs were recovered at the end point (12 weeks after BLT surgery) from the tissues of sets 1 and 2 mice, respectively (Fig. 2D).

Given the semirandomness of lentiviral IS selection and the large human genome (3 billion bases), the likelihood of identical IS to occur with different vectors or different animals is negligible. We did, however, find that 51 ISs (7%) and 454 ISs (8%) in the 454-pyrosequencing and MiSeq datasets, respectively, were “collision” events (or sequence crossovers): In other words, the identical IS appeared in more than one vector (H1 or H5) or the HIV-1 IS sequence group (Fig. 2, B and D). These IS sequence collisions are a common problem for modern high-throughput sequencing (*34*, *45*), likely occurring due to sample cross-contamination or demultiplexing errors (e.g., sequencing errors or mutations within the H1 and H5 index sequences). In contrast to our previous conventional (non–LTR indexing) IS analysis, where EGFP^+^ and mCherry^+^ flow-sorted cell pools were used for sequencing (fig. S3), the new LTR indexing approach does not require prior cell sorting and thus eliminates any potential sample cross-contamination that may occur during the cell-separation processes. The LTR indexing approach showed approximately 3.1- to 3.6-fold lower collision rates than those of conventional, non–LTR-indexing IS analysis (Fig. 2D).

LTR index read errors occurred at a rate of 1.92 and 0.73% for sets 1 and 2 individual sequences, respectively (fig. S4A). LTR index collisions can be effectively identified and corrected when identical IS sequences are available for the purpose of index sequence comparison (fig. S4B). A commonly used procedure for handling such collisions is to identify the “correct” IS by choosing the most frequent IS, that is, one showing a ≥10-fold higher detection frequency than any of the others (*35*, *36*). The average IS frequency differences between correct and incorrect IS were 92-fold (±160 SD) and 192-fold (±157 SD) in the 454-pyrosequencing and MiSeq datasets, respectively (Fig. 2C). After applying these criteria, only five ISs (0.7%) in the first set (454-pyrosequencing data) and none in the second set (MiSeq dataset) remained unresolved, reflecting the higher sequencing depth of the MiSeq dataset (Fig. 2D). These unresolved ISs were excluded from the clonal profile analysis. The low-copy IS clones that did not show any LTR index collisions remained in our final data. There remains, however, a low level of uncertainty in LTR index identities for these low-copy IS clones: e.g., about 1.92 and 0.73% (or less) uncertainty due to potential read errors.

### Multiplexed IS analysis using LTRi-Seq reveals polyclonal HSPC repopulation and HIV-1–mediated selection for anti-HIV–modified clones in infected mice

**Fig. 3:**
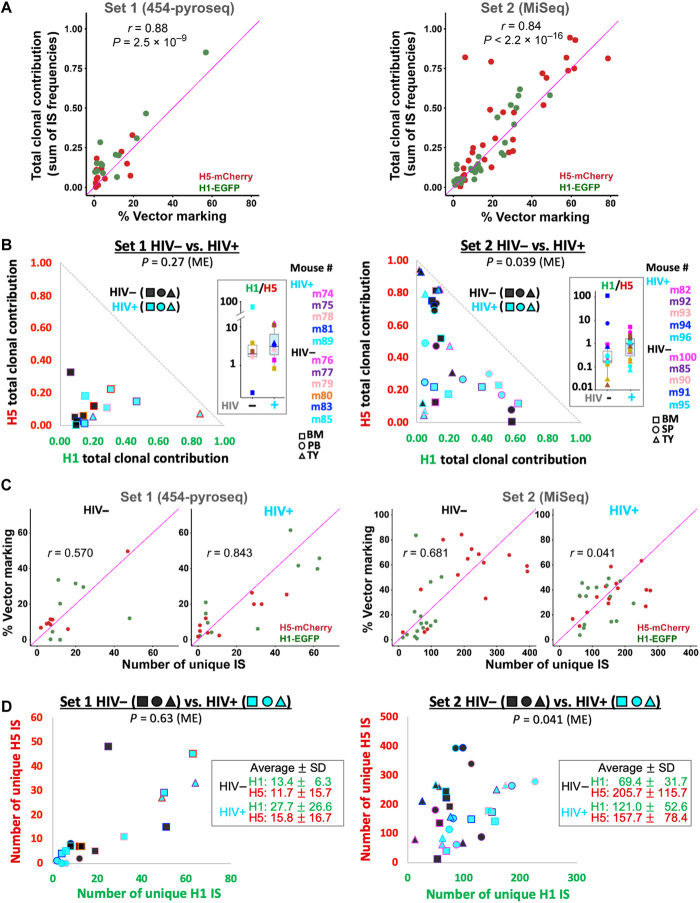
HIV-1–induced selective clonal expansion of anti-HIV vector–transduced cells. (**A**) A direct comparison of the total IS clonal contribution (sum of all IS frequencies, *y* axis) for H1-EGFP-dual-shRNA (H1-EGFP; green dots) and H5-mCherry clones (red dots) versus the corresponding EGFP or mCherry gene markings in CD45^+^ cells (flow cytometry results shown as % vector marking, *x* axis) from the same organ samples at the 12-week end point. Data indicate a strong correlation between the two parameters [Pearson’s *r* = 0.88 for set 1 (left) and *r* = 0.84 for set 2 (right)]. The diagonal line is a reference line (*r* = 1). (**B**) The two-dimensional (2D) plots showing the total combined contribution of H5 IS clones (*y* axis) and H1 IS clones (*x* axis) in HIV+ organs (cyan squares, triangles, or circles) and HIV− organs (black squares, triangles, or circles), including BM (squares), TY (triangles), PB (circles in set 1), and SP (circles in set 2). The outline colors of the squares, triangles, and circles correspond to the various mouse IDs. H1/H5 ratios are shown in the box. The *P* values were calculated using mixed-effects gamma regressions (ME) (fig. S8B). (**C**) The number of unique IS recovered from each sample was also positively correlated to the vector marking level of the sample. Scatter plots show a correlation between the number of total unique IS recovered from each organ sample (*x* axis) and respective EGFP or mCherry markings in CD45^+^ cells from the same organ samples (*y* axis). (**D**) Polyclonal repopulation by both H1-EGFP– and H5-mCherry–engineered cells. The 2D plots show the total number of unique IS of H1- (*x* axis) and H5-marked (*y* axis) cells in different organ samples. The shape and line color strategies are identical to those in (B). The average number of H1 IS clones increased approximately twofold in HIV+ samples relative to that in HIV− samples, while H5 IS clone numbers remained relatively constant.

We compared IS sequence data with EGFP and mCherry gene-marking data shown above. To better present the frequencies of individual IS clones relative to the total repopulating cell pool, composed of both vector-marked (H1 and H5) and unmarked cells, we used IS clonal contribution data that factor in the % unmarked cells (see Materials and Methods for more details). The total combined IS clonal contribution for anti-HIV and control vectors showed a positive correlation with the marking levels of EGFP and mCherry in CD45^+^ cells (the Pearson correlation coefficients *r* = 0.88 and *r* = 0.84 for the first and second sets, respectively) (Fig. 3A). Similar positive trends between IS data and flow cytometry data have been reported in nonhuman primate studies, showing normal polyclonal reconstitution of HSPCs (*32*). The baseline H1 anti-HIV and H5 control sequence ratios in HIV– mice differed from one set of experiments to the other, but both sets showed increased H1 clonal contribution and H1/H5 ratios in infected mice when compared with baseline H1/H5 ratios in HIV– mice, indicating a selective expansion of HIV-protected cells in infected mice (Fig. 3B). Mixed-effects gamma regressions analysis comparing the H1/H5 ratios in HIV and HIV+ mice showed *P* = 0.039 for set 2 but, despite notable differences, set 1 showed *P* = 0.27, possibly due to the relatively low number of organ samples available for the set 1 analysis.

The number of unique IS recovered for these two vectors also reflected EGFP and mCherry markings (Fig. 3C). The HIV+ mice showed greater IS recovery rates for H1 anti-HIV–protected cells than did HIV– mice, again indicating the potential selective advantage of HIV-protected cells (Fig. 3D). Mixed-effects gamma regressions comparing H1/H5 ratios in HIV+ and HIV− mice showed *P* = <0.001 for set 2, but set 1 showed *P* = 0.63, probably for the same reasons addressed above. Notably, there was no significant reduction in the average number of unique IS for the control clones in the HIV+ animals; the unique IS showed a 1.3-fold increase in the first set and a 0.8-fold decrease in the second set when compared with those in HIV– animals. This observation indicates that polyclonal repopulation by control H5-mCherry HSPCs occurs even in the presence of HIV-1 infection, in turn suggesting that, while HIV-1–mediated selection of the anti-HIV–modified cells appears to be evident in infected mice, the impact of HIV-1_NFNSX_ infection on HSPC BM homing and polyclonal hematopoiesis was insignificant in our humanized mouse study.

### Insubstantial impact of HIV-1 infection on polyclonal HSPC hematopoiesis and tissue repopulation

**Fig. 4:**
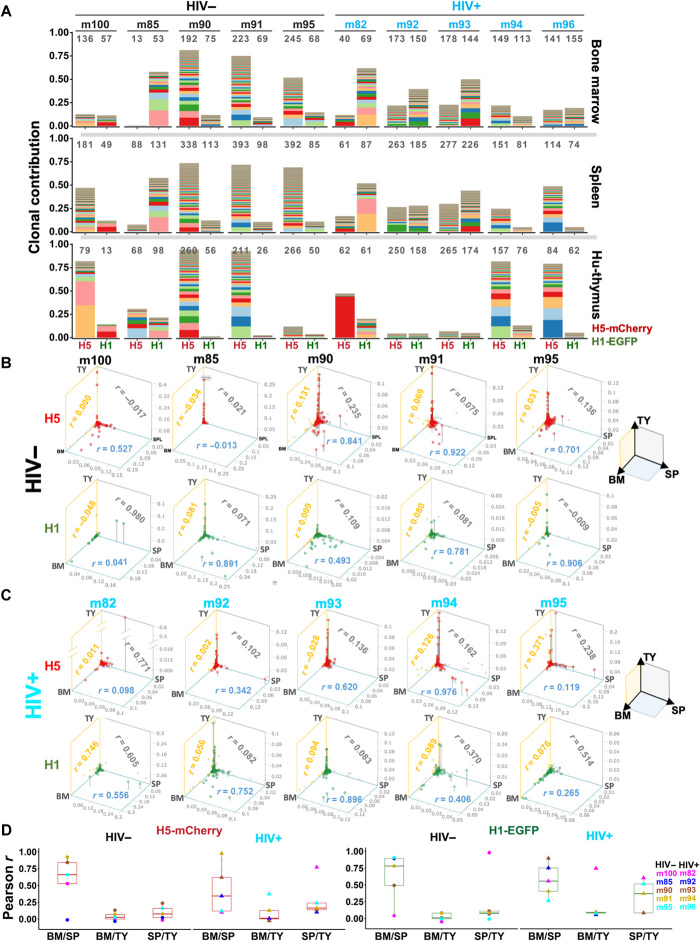
HIV-1 infection has no significant impact on clonal size distribution or clonal repopulation in different organs. (**A**) Stacked bar plots show the relative clonal contributions (*y* axis) of H5-mCherry clones (H5) and H1-EGFP-dual-shRNA clones (H1) in the BM, SP, and human TY of HIV− and HIV+ mice from set 2. Each color band in a stack represents individual IS clones, and its thickness corresponds to the relative clone frequency. Clones are stacked in a descending order of frequency with the highest frequency clone at the bottom and lowest to the top. The total number of IS clones (gray) and mouse IDs (black or blue) are shown at the top the chart. (**B** and **C**) 3D scatter plots show H5-mCherry (H5; red dots) and H1-EGFP-dual-shRNA clonal frequencies (H1; green dots) in BM, SP, and TY of HIV− mice (A) and HIV+ mice (B). Each red or green dot represents the individual IS clones of H1-EGFP and H5-mCherry cells, respectively. These dots are positioned in 3D space based on IS clonal frequencies in BM, SP, and TY as three coordinates. Pearson’s *r* values comparing the clonal profiles of BM and TY, BM and SP, and TY and SP are shown on the yellow, blue, and gray panels, respectively. (**D**) Boxplots of Pearson’s *r* values between BM and SP (BM/SP), BM and TY (BM/TY), and SP and TY (SP/TY) for H5-mCherry (left) and H1-EGPF-dual-shRNA clonal profiles (right) in HIV− and HIV+ mice.

We next analyzed organ-to-organ clonal distributions in both HIV+ and HIV– mice of set 2 (Fig. 4). Set 1 was excluded because of the lack of essential organ data (fig. S5). When we compared the H5 (control mCherry^+^ cell) IS profiles of BM, spleen, and human thymic implant of each HIV– mouse, we found a unique organ-to-organ repopulation pattern (Fig. 4, B and D). BM and spleen showed relatively similar clonal profiles (average Pearson correlation coefficient *r =* 0.587 ± 0.369 SD), whereas the correlation was poorer when thymic organelle was compared with BM or spleen (average *r* = 0.038 ± 0.063 and *r* = 0.094 ± 0.103 SD, respectively). Similar organ-to-organ IS distribution patterns were observed for the H1 anti-HIV vectors in HIV− mice (Fig. 4B) and for both H5 and H1 in HIV+ mice (Fig. 4C). The observed organ-to-organ IS patterns, in which some of clones were notably expanded only in the thymic organelle, may reflect the unique clonal behaviors of thymocytes resulting from transient and extensive clonal expansion during normal T cell development, which manifest in unique clonal profiles in the thymic organelle, as previously demonstrated by Brugman *et al.* (*46*).

To further address the impact of HIV-1 infection on HSPC function, we analyzed intra-organ clone-size variations. In a previous mathematical modeling study, we demonstrated that variations in the shapes of the clone-size distribution of blood repopulating cells reflect differences in the number of engrafted HSPCs and their functional parameters (birth, death, and differentiation rates) (*47*). All H1 anti-HIV and H5 control repopulating cells in the second set of animals showed highly variable IS clonal sizes (Fig. 4, A to C). When the shapes of clone-size distributions were compared, as described previously (*47*), all anti-HIV and control cells, particularly in the spleens of both HIV+ and HIV– mice, showed similar shapes (fig. S6A). The observed clone-size patterns also mirrored those of the blood repopulating cells of rhesus macaques (*32*, *47*). To further study the impact of HIV infection on clonal distribution, we used Rényi diversity profiles (*48*) (details in Materials and Methods). Rényi diversity plots show sloped diversity profiles in all organs, and the overlapping of Rényi diversity curves from both HIV+ and HIV− mouse samples (fig. S6B) signifies no particular ordering of clonal diversities, likewise suggesting that HIV-1 infection has no significant impact on normal clonal repopulation. These data thus consistently point to the efficient homing, hematopoiesis, and tissue repopulation of both HIV-protected and control HSPCs, even in the presence of HIV-1 infection, as demonstrated in our humanized mouse model.

### HIV-1 proviral IS patterns are distinct from those of lentiviral vectors

In parallel with the IS of the two lentivectors (anti-HIV and control), a total of 111 and 1144 HIV-1 proviral DNA ISs were recovered in our first and second sets of experiments, respectively (see Fig. 2D). These HIV-1 ISs account for approximately 15.3 and 19.9%, respectively, of all the unique ISs (lentiviral vectors and HIV-1) recovered in each set. However, the total combined HIV-1 IS sequences constituted only 3.2% of all the IS sequences in set 1 and 2.1% in set 2, respectively, reflecting considerable frequency differences between the HIV-1–infected and lentiviral vector–engineered cell clones. Although much smaller, on average, than lentiviral vector clones, individual HIV-1 IS clones also showed varying levels of detection frequencies. Of the 1144 HIV-1 unique ISs recovered from three different organs (BM, spleen, and human thymic implant) in the second set animals, 77 (6.7%) were recovered in at least two different organs.

**Fig. 5:**
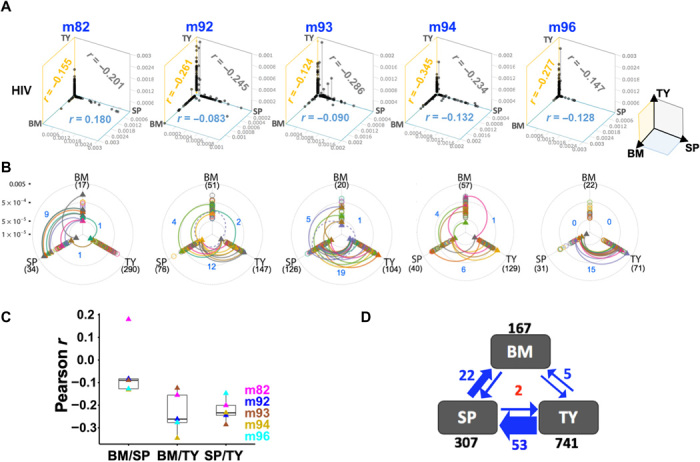
Organ-to-organ distribution of HIV-1 provirus IS clones distinct from that of lentiviral vectors. (**A**) 3D scatter plot shows HIV-1 IS clones (black dots) and their relative contributions (IS frequencies) in the BM, SP, and human TY of five infected mice. Pearson *r* values comparing the IS profiles of BM and TY, BM and SP, and TY and SP are shown on the yellow, blue, and gray panels, respectively. (**B**) The frequency differences among the common HIV-1 IS detected in multiple organs are shown. The relative frequencies of all IS clones recovered in BM, SP, and TY were plotted on BM, SP, and TY axes, respectively. Those ISs common to two different organs, linked by colored lines, showed significantly different frequencies in two comparative organs. The two IS clones detected in all three organs are shown in dash lines. The total number of unique IS for each organ is shown in a bracket. (**C**) Boxplots comparing Pearson’s *r* values for the HIV-1 IS profiles of two organs. (**D**) Collective HIV-1 IS data from all infected mice. The number of IS common to two comparative organs is shown in blue. The arrows represent the quantities of common IS in the two comparative organs, with the direction of the arrows indicating the direction of higher to lower sequence frequencies of the IS common to the two organs. The two IS found in all three organs are in red. The black numbers indicate the total quantities of recovered IS for each organ.

A two to fourfold higher number of HIV-1 ISs were recovered in the human thymic implant (average 148 ISs, ±84 SD) than in BM (33.4 ISs, ±19 SD) or spleen (61.4 ISs, ±40.4 SD). Overall, we found a consistent and unique organ-to-organ IS pattern, distinct from lentiviral vector IS patterns, in which the HIV-1 IS clones that were detected at a high frequency in one organ were undetectable or detected at a much lower frequency in any of other organs, resulting in poor statistical correlations in organ-to-organ IS comparison for all combinations of organs tested (average *r =* −0.050 ± 0.131 SD to −0.233 ± 0.091 SD) (see Fig. 5, A and C). This pattern suggests the limited proliferation and organ-to-organ circulation of HIV-1–infected cell clones. For example, reflecting the development and circulation of T cells in various lymphoid organs, some of the high-frequency IS clones of the thymic organelle were detectable in the spleen at a lower frequency, whereas other high-frequency clones of spleen or BM were only rarely detectable in the thymic organelle (Fig. 5, B and D). Our data suggest that HIV-1–infected human thymocytes may clonally expand and migrate to other organs, whereas HIV-1–infected BM or spleen cells (mature T cells mostly) are not imported back to the thymic organelle. It is statistically highly unlikely that these organ-to-organ HIV-1 IS distribution patterns are simply the result of random contamination or sequencing errors (fig. S7). Our data thus provide novel insights into the limited circulation/migration abilities of HIV-1–infected cells in different body organs, suggesting that the pathologic impacts of HIV-1–infected clones may likewise be locally limited and confined, at least for the short term.

## DISCUSSION

In this study, the potential effects of HIV-1 infection on HSPC transplant, survival, and organ repopulation were investigated using HIV-1–preinfected, humanized mice. LTRi-Seq enabled effective and unbiased analysis of two lentiviral vectors (anti-HIV H1-EGFP-dual-sh1005/sh516 and control H5-mCherry vectors) and HIV-1 proviruses. Our data provide novel insights into the behaviors of HSPC and HIV-1–infected cell clones in vivo, insights with important implications for the repopulation of HSPC in the context of HSPC transplant and genetic therapy for HIV-infected patients.

We cotransplanted both H1 anti-HIV–modified and H5 control HSPC pools into the same host to better evaluate anti-HIV modifications in BLT mice, where host-to-host and experiment-to-experiment variations are common (*38*, *43*). The analysis of vector IS in this type of competitive repopulation assay is challenging, particularly when testing lentiviral vectors in the presence of HIV-1 infection, due to the common LTR sequences shared by the therapeutic vectors and HIV-1 proviruses. LTRi-Seq enabled simultaneous analysis of anti-HIV vector (H1 indexed)– and control vector (H5 indexed)–marked HSPC clones and HIV-1–infected cell clones (wild-type LTR) in the same mice. With the new approach, we have evaluated whether anti-HIV– and control-engineered HSPC would efficiently engraft and repopulate the blood system in the presence of HIV-1 infection. ISs were sequenced and analyzed on the basis of methods proven effective in quantifying repopulating clones (*32*, *44*).

Here, we demonstrate polyclonal and normal tissue repopulation of both types of HSPCs using our unique HIV-1 preinfection mouse model. By our analysis, we showed that HIV-1–mediated selection primarily occurred at the mature T helper cell level and not significantly at the HSPC level. In clonal profile analysis, a complete absence of differences between HIV+ and HIV− samples is not expected even if the HIV-1 infection has had no effect on HSPC; a small portion of mature cells will still be killed in infected mice. However, if HIV-1 infection affected HSPC homing and repopulation, then there would be notable differences in the clonal profile analysis. While a selective advantage for the H1 anti-HIV clones was evident, the control H5 cells (those with no anti-HIV modification) showed similarly high levels of polyclonal engraftment in both HIV+ and HIV– mice. Despite the known limitations of xeno-transplant models (*38*, *43*, *49*), this polyclonal engraftment in infected hosts is noteworthy given the numerous previous reports on the direct and indirect effects of HIV infection on BM niche cells, including HSPC (*15*), stromal cells (*16*), and possibly, CD4^+^ T cells that reside in the niche (*17*). It appears that, following infusion, both types of HSPCs must have competed normally for the available HSPC niches, which remained functionally normal even in infected hosts, and HIV-1 infection had minimal impact on the polyclonal hematopoiesis and tissue repopulation of transplanted HSPCs.

Our data have important implications for the repopulation of gene-modified HSPC in the context of gene therapeutic clinical studies for the treatment of HIV-1 diseases. Our study is the first to experimentally evaluate the selective engraftment and clonal repopulation of gene-modified HSPCs in the presence of HIV-1 infection using a new preinfected mouse model. The premise of anti-HIV gene therapy is that anti-HIV gene engineering of a patient’s own HSPC can result in the selection of HIV-protected cells during hematopoietic reconstitution, long-term control of viral replication, and a favorable clinical course leading to a “functional cure.” Notably, virtually all of the T cells in the Berlin and London patients were replaced with *CCR5*∆*32*/∆*32* donor cells and cleared virus (*8*, *9*, *50*). Past anti-HIV clinical gene therapy trials, by contrast, have failed to demonstrate any clinical benefit in patients, primarily due to the scarcity of gene-engineered cells repopulating in the blood (*10*–*13*). Even patients who have received myeloablative preconditioning, a treatment that significantly improves HSPC engraftment, have shown less than 0.32% anti-HIV gene marking in their peripheral blood mononuclear cells (*11*). Most salient among the many possible explanations for the low gene marking in patients with HIV is the possible impairment of HSPC function by direct HIV-1 infection and/or by an HIV-damaged BM microenvironment (*15*, *16*). However, most in vivo preclinical studies so far have tested HSPC transplant in the absence of HIV-1 infection and only subsequently challenged the repopulating mature cells with HIV-1 to evaluate anti-HIV gene modification (*20*–*26*). Recent nonhuman primate studies, testing HSPC transplant in SHIV-infected, ART-suppressed animals, have identified perturbations in the immune system following irradiation therapy (*14*, *18*, *19*). Our data demonstrate that the HIV-mediated selection for anti-HIV–modified cells was limited to a portion of mature cells, and the effects of viral infection on HSPC’s homing and organ repopulation were insubstantial in our humanized mouse study.

Our study showing the normal polyclonal HSPC repopulation in the presence of HIV-1 infection supports the use of planned cART interruption during HSPC transplant. Although cART interruption has been recommended in recent studies (i) to minimize therapy-related toxicity and (ii) to improve anti-HIV gene marking in the peripheral blood, the safety and efficacy of cART interruption remain unclear and controversial (*2*, *3*, *14*, *19*). Recent human and nonhuman primate studies have shown that autologous HSPC transplant in HIV-1–infected patients is well-tolerated and feasible (*4*, *7*, *18*), but these studies evaluated HSPC transplant in the presence of cART and lacked a clonality analysis of the repopulating cells.

Furthermore, using LTRi-Seq, we have characterized HIV-1–infected cell clones in comparison to repopulating HSPC clones. Clonal expansion of HIV-1–infected cells has recently been reported in humans and humanized mice and is suspected to be an important mechanism of HIV-1 persistence (*51*). The clonal dynamics and organ-to-organ distribution of expended clones, however, remain poorly investigated. Our data demonstrate a unique pattern of organ-to-organ clonal profiles characteristic of HIV-1–infected cells, distinct from the clonal patterns of gene-engineered HSPC clones, potentially suggesting strong organ confinement and limited circulation for at least 15 weeks after infection. A recent human case study, by contrast, showed wide anatomic distribution of a few infected clones, likely the result of the effects of years of HIV-1 infection and cancer in the patient (*52*). A recent humanized mouse study analyzing HIV-1–infected cell clones at 15 weeks after infection has shown clonal patterns consistent with our results, identifying only a small fraction of HIV IS common in multiple organs (*53*).

Preinfection BLT mice are a practical and functional small-animal model with which to directly test therapeutic vectors in the presence of HIV-1 infection. In vitro experiments ignore the impact of complex tissue architecture, while in vivo studies in humans do not permit adequate experimental manipulation. Nonhuman primate models require the use of modified simian-version viruses. Both sets of our BLT mouse experiments have reproducibly demonstrated consistent polyclonal HSPC engraftment in all test animals and for all vector types. The clone-size distribution patterns, in particular, closely resemble those observed in our previous nonhuman primate studies of autologous HSPC transplant (*32*, *47*), indicating the potential relevance of our data for HSPC transplant in HIV-infected individuals.

IS sequencing–based, lentiviral tagging approaches have been widely used to study HSPC clonal repopulation, and the data analytic procedures are well established (*30*–*36*). Unlike other lentiviral tagging approaches that use short synthetic barcodes to distinguish among different cellular clones, IS sequencing approaches directly compare tagged sequences to the reference human genome and thereby enable highly accurate IS clone identification even for single-copy IS events (*27*, *54*). In our study, IS clones and LTR indexes were determined by independent procedures. We found LTR index collision events in about 7 and 8% of IS clones due to read errors for LTR index sequences (average 1.92% for set 1 and 0.73% for set 2), but most of these index read errors can be readily identified and are correctable. Only 0.7% in set 1 and none in set 2 IS clones remained uncorrected after applying our 10× correction criteria; these were removed from clonal profile analyses. The low-copy IS clones, those that are not showing collision events, remaining in the final clonal profile data have approximately 1.92 and 0.73% (or less) uncertainty in their LTR index identities due to the potential read errors. The application of a threshold for low reads (*27*) would remove these uncertainties, but needs to be carefully applied as this would remove a significant amount of bona fide low-copy data.

In summary, our LTRi-Seq data provide new information on the repopulation of transplanted HSPCs in the presence of HIV-1 infection and the clonal profiles of HIV-1–infected cells in key lymphoid organs. These results are particularly relevant to the issue of ART interruption in the context of HSPC transplant and anti-HIV gene therapy for HIV-infected individuals. The concepts and technological tools arising from this study will be critical for the development of future gene therapy protocols.

## MATERIALS AND METHODS

### Lentiviral vector construction and production

The H1-EGFP-dual-shRNA (sh1005/sh516) and H5-mCherry lentiviral vectors were derived from the FG12-sh1005/sh516 (*21*) and FG12-mCherry lentiviral vector (*20*), respectively, by introducing point mutations at the 3′-end of the left LTR. The primers used for H1 were H1f (5′-CAGTGTGGAAAATCTCCAACAGTGGC) and H1r (5′-TGTTCGGGCGCCACTGTTGGAGATTT), and the primers for H5 were H5f (5′-TGGAAAATATCCAACAGTGGCGCCCGAACAG) and H5r (5′-CTGTTCGGGCGCCACTGTTGGATATTTTCCA). All lentiviral vectors were produced by the calcium phosphate–mediated transient transfection of 293T cells described previously (*20*, *21*, *55*). Briefly, 293T cells, cultured in Dulbecco’s modified Eagle’s medium with 10% fetal calf serum, penicillin (100 U/ml), and streptomycin (100 μg/ml), were transfected with vesicular stomatitis virus glycoprotein (pHCMV-G), packaging (pCMVR8.2DVPR), and lentiviral vector plasmids. Virus supernatant was collected on days 2 and 3 after transfection, filtered through a 0.22-μm pore-size filter and concentrated 100-fold by ultracentrifugation. Lentiviral vector stocks were titrated by infecting 293T cells (10^5^) with various dilutions of the concentrated virus stock; this was followed by flow cytometry analysis of EGFP or mCherry expression 3 days after infection.

### Humanized mice and HIV-1 infection

All the mouse experiments and HIV-1 infection procedures are described in detail in our previous publication (*39*). Briefly, neonatal (1 to 3 days old) nonobese diabetic.Cg-*Prkdc^scid^Il2rg^tm1Wjl^*/SzJ (NSG) mice were irradiated (125 centigrays) and transplanted with human fetal liver CD34^+^ HSPCs by intrahepatic injection; after 11 weeks, half of the mice were infected with CCR5-tropic HIV-1NFNSX (200 ng of p24 Gag). After 3 weeks of infection, both HIV+ and HIV− mice were myeoablated with busulfan (35 mg/kg) and, the next day, transplanted with a piece of thymus and an equal mixture of H1-EGFP-dual-shRNA and H5-mCherry vector–transduced CD34^+^ HSPCs via a two-step procedure (implantation of the Matrigel-solidified CD34^+^ cell mix and infusion of the gel-free cell mix through the retro-orbital vein plexus on the same day). Human CD34^+^ HSPCs isolated from fetal livers and thymus pieces from the same donor were cryopreserved, as previously described (*39*). Human fetal thymus and fetal livers were obtained from Advanced Bioscience Resources, FPA Women’s Health, and the University of California, Los Angeles (UCLA) Center for AIDS Research (CFAR) Gene and Cellular Therapy Core. The UCLA Institutional Review Board has determined that fetal tissues from diseased fetuses obtained without patient-identification information are not human subjects. Written informed consent was obtained from patients for the use of the tissue for research purposes. All mice were maintained at the UCLA CFAR Humanized Mouse Core Laboratory in accordance with a protocol approved by the UCLA Animal Research Committee. Flow cytometry and the viral load assay have been described in detail in a previous publication (*39*).

### IS sequencing analysis

For IS sequencing, we followed the procedures described in our previous publication (*32*, *44*). In this study, we focused on analyzing only the right LTR junctions. Briefly, 1 mg of genomic DNA for set 1 samples and 2 mg of genomic DNA for set 2 samples, with a few exceptions (see tables S1 and S2), were subject to a linker-mediated PCR method using RsaI and CviQI restriction enzymes. Linker-ligated IS DNA fragments were amplified by a nested PCR strategy using two LTR primers, 1R-primer (5′-CTGGCTAACTAGGGAACCCACT-3′) and 2R-primer (5′-ACTCTGGTAACTAGAGATCC-3′) that align 140 and 57 bases upstream of the 3′-end of U5 LTR, respectively, and two primers that align on linker DNA. This strategy ensured that both the LTR indexes and vector-host junctions originating from cell clones could be PCR amplified and sequenced without any primer-associated bias. Set 1 samples were sequenced with Roche 454-pyrosequencing (Roche FLX genome sequencer) and set 2 samples were sequenced with a Illumina MiSeq sequencer. To process the 454-pyrosequencing data, we used a previously described python script (*32*, *44*) with the additional feature of enabling identification and separation of IS sequences based on the LTR index sequence. Sequences that included both the 3′-end U5 LTR DNA and ≥25-base host DNA (with ≥95% homology to the human genome) with the 3′-end LTR sequence at the virus-host junction were considered a true IS sequence. Briefly, sequences that showed the 3′-end LTR sequence joined to genomic DNA were identified using the Smith-Waterman algorithm [for 454 data, Emboss water tool http://emboss.sourceforge.net/ and for Illumina data, a modified version of SSW library (*56*) in C++] and then further tested for the presence of LTR indexes (H1, H5, or wild type). Genomic sequences shorter than 25 bases were removed. On the basis of the LTR index, IS reads were assigned to either H1 and H5 vectors or WT HIV-1. We followed similar procedures for the analysis of Illumina MiSeq data. IS sequences were mapped onto the human genome (Version hg19 downloaded from https://genome.ucsc.edu/) using either BLAT (BLAST-like alignment tool) for 454 data or Burrows-Wheeler Aligner software (*57*) for Illumina data. Sequence-mapping and IS-counting procedures were identical to those in our previous publications (*44*). To better present the IS clonal frequencies relative to the total repopulating cell pool, we calculated IS clonal contributions that factor in the % unmarked cells. The relative sequence frequencies of individual IS, initially normalized by the total count of H1 and H5 IS sequences combined, were multiplied by the fraction of total vector marked cells (both EGFP^+^ and Cherry^+^ cells combined) in CD45^+^ cells. This approach enabled direct comparison of IS data to flow cytometry gene marking data and direct comparison of IS clones in different organs.

### Signal crossover correction

Because of the semirandom nature of vector/virus integration into the host genome, events in which the same IS appeared in multiple animals or in different LTR index datasets were considered collisions or “signal crossovers.” To identify the correct and incorrect sample identities among the same IS collision events for both 454 and Illumina data, we used a commonly used criterion that identifies sequencing errors (or sample contamination) based on sequence count differences among the same IS collision events (*34*–*36*). Correct sample identities were established when a sequence count was at least 10 times higher than the counts of all of the others in the dataset sharing the same IS (see Fig. 2C and fig. S4). IS data with an incorrect sample identity were removed from the dataset. Any signal crossover IS events that failed to show >10-fold sequence count differences were considered “unresolved” and removed from the dataset.

### Statistical analysis

Student’s *t* tests with Welch correction were used to compare continuous variables between pairs of experimental conditions, including the levels of cell populations and the H1/H5 ratios between HIV− and HIV+ humanized mice. An exact Mann-Whitney test was used to compare organ-to-organ IS crossover rates within the same animal. Pearson correlation (*r*) was used to compare pairs of continuous variables, including total clonal contribution versus % vector marking and number of unique IS versus % vector marking; clonal frequencies within pairs of organs (BM, spleen, and thymus) in HIV− and HIV+ mice; and IS profiles within pairs of organs in HIV+ mice. The randomness of organ-to-organ IS distribution patterns was evaluated by three pair-wise χ^2^ tests (fig. S7). For example, we tested whether the overlap of IS expressions with BM differed between SP and TY tissue types. A similar analytic framework was used for the other tissue pairs (spleen and BM and thymic organelle and BM). To evaluate the impact of HIV-1 infection on anti-HIV vector (H1-EGFP-dual-shRNA)– and control vector (H5-mCherry)–transduced cells (Fig. 1D and Fig. 3, B and D), we used mixed-effects gamma regression models with a log link and unstructured covariance matrix to compare cell% and IS frequency between H1 and H5 in HIV+ and HIV− mice, adjusting for tissue type. Gamma regression was chosen over linear regression because of distribution skew among the cell% and IS frequencies. The models included an interaction term between H1 (versus H5) and HIV status to test whether the effect of H1/H5 differed by HIV status. For cell% and IS%, when the outcome measure was 0, a small number (0.1) was added to meet the range requirement of gamma regression. *P* values were reported from the models. Statistical significance was assessed at the 0.05 level, and analyses were implemented in R v.3.4.4 (*58*). More details on statistical analysis can be found in data file S1.

### Clonal diversity analysis

We investigated the impact of HIV-1 infection on the diversity of total clonal repopulation in different organs of HIV− and HIV+ mice using Rényi’s diversity profiles. The *y* axis of the Rényi’s diversity plot, ***H***_**α**_, indicates species diversity, such that consistently higher values of ***H***_**α**_ indicate a more diverse clonal sample. The *x* axis is **α**, which ranges from 0 to infinity (*59*). If the lines or profiles for two groups cross, then their relative diversities are unknown. Diversity plots also indicate the evenness of the clones, where a horizontal line indicates equal expansion of each clone (i.e., uniform clonal expansion), and steeper slopes indicate greater nonuniform clonal expansion [see Kindt *et al.* (*60*), chapter 5 page 56]. Details of Rényi’s diversity calculation are in Supplementary Text.

## Supplementary Material

aay9206_SM.pdf

aay9206_Data_file_S1.xlsx

## SUPPLEMENTARY MATERIALS

Supplementary material for this article is available at http://advances.sciencemag.org/cgi/content/full/6/30/eaay9206/DC1

## Appendix

View/request a protocol for this paper from *Bio-protocol*.
