# Supplementary material for: The clonal repopulation of HSPC gene modified with anti–HIV-1 RNAi is not affected by preexisting HIV-1 infection
Source: Sci Adv. 2020 Jul 22;6(30):eaay9206. doi: 10.1126/sciadv.aay9206 (PMC7385479; doi:10.1126/sciadv.aay9206)
Supplement: aay9206_SM.pdf [file aay9206_SM.pdf]

Science Advances
AAAS

advances.sciencemag.org/cgi/content/full/6/30/eaay9206/DC1

Supplementary Materials for

The clonal repopulation of HSPC gene modified with anti–HIV-1 RNAi is not affected by preexisting HIV-1 infection

Gajendra W. Suryawanshi, Wannisa Khamaikawin, Jing Wen, Saki Shimizu, Hubert Arokium, Yiming Xie, Eugene Wang, Shihyoung Kim, Hyewon Choi, Chong Zhang, Hannah Yu, Angela P. Presson, Namshin Kim, Dong-Sung An, Irvin S. Y. Chen*, Sanggu Kim*

*Corresponding author. Email: syuchen@mednet.ucla.edu (I.S.Y.C.); kim.6477@osu.edu (S.K.)

Published 22 July 2020, *Sci. Adv.* **6**, eaay9206 (2020)
DOI: 10.1126/sciadv.aay9206

The PDF file includes:

Figs. S1 to S8
Tables S1 and S2

Other Supplementary Material for this manuscript includes the following:

(available at advances.sciencemag.org/cgi/content/full/6/30/eaay9206/DC1)

Data file S1

Supplementary data

Supplementary Figure S1 (a)

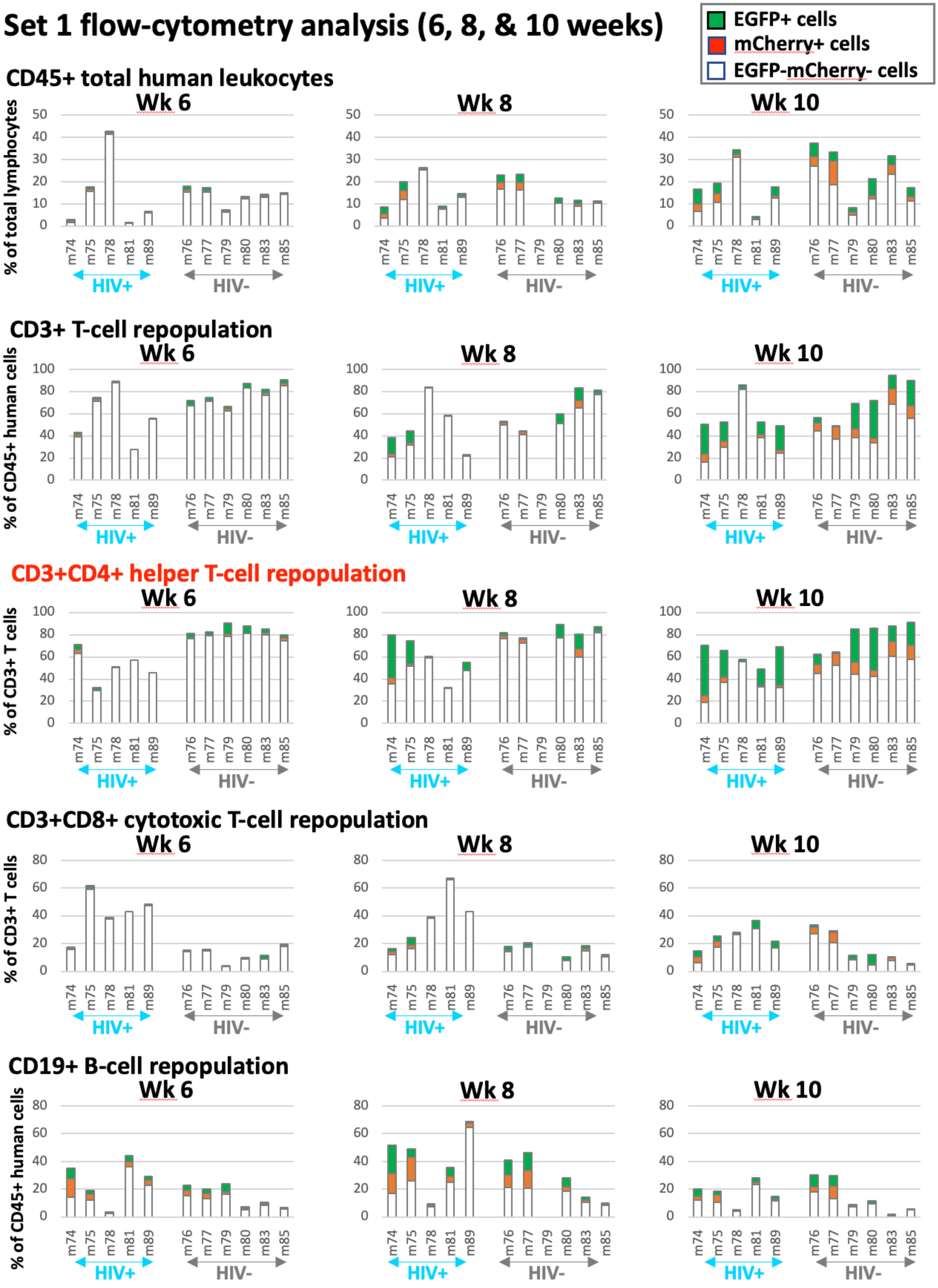

Supplementary Figure S1 (b)

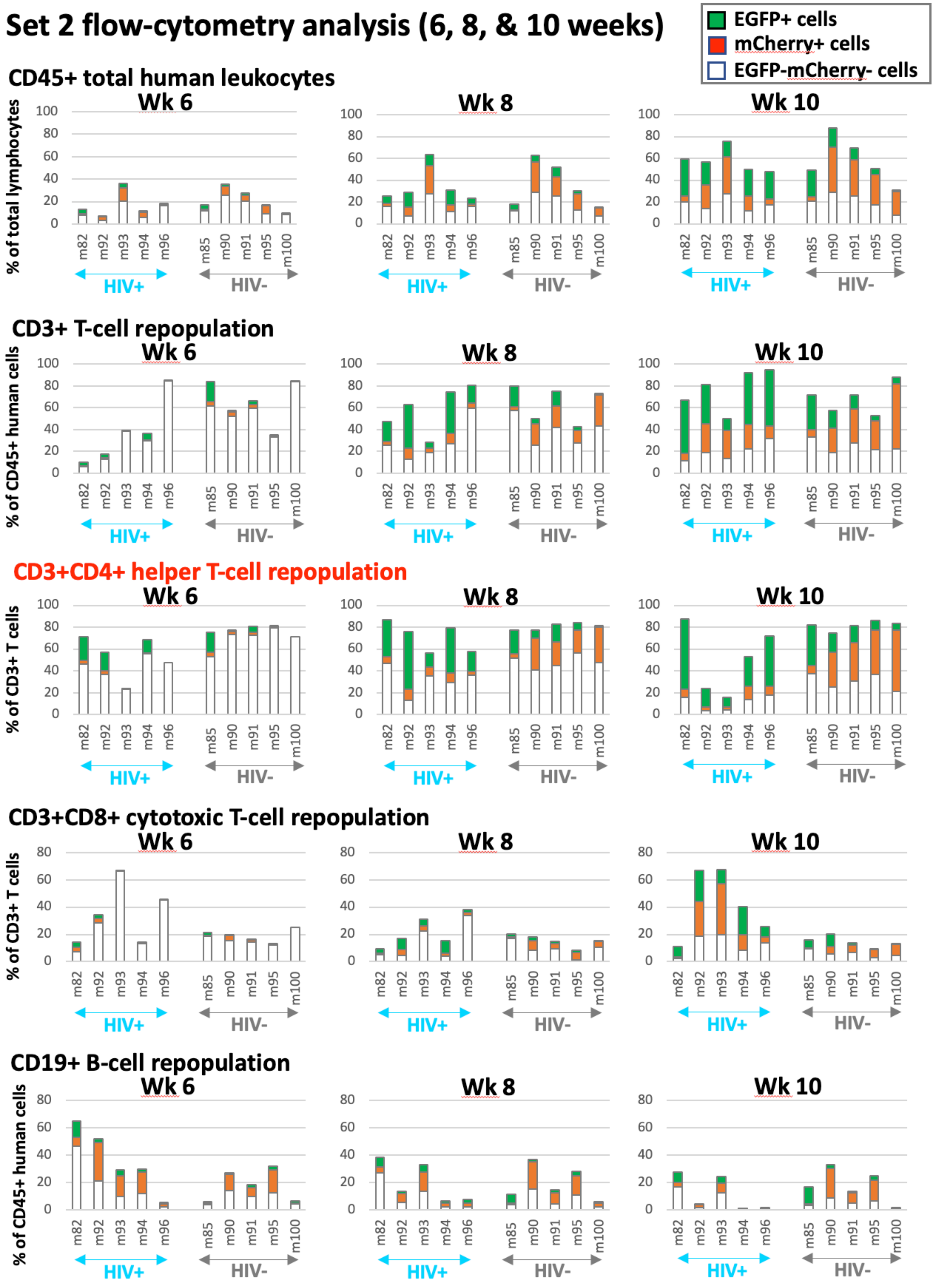

Supplementary Figure S1 (c)

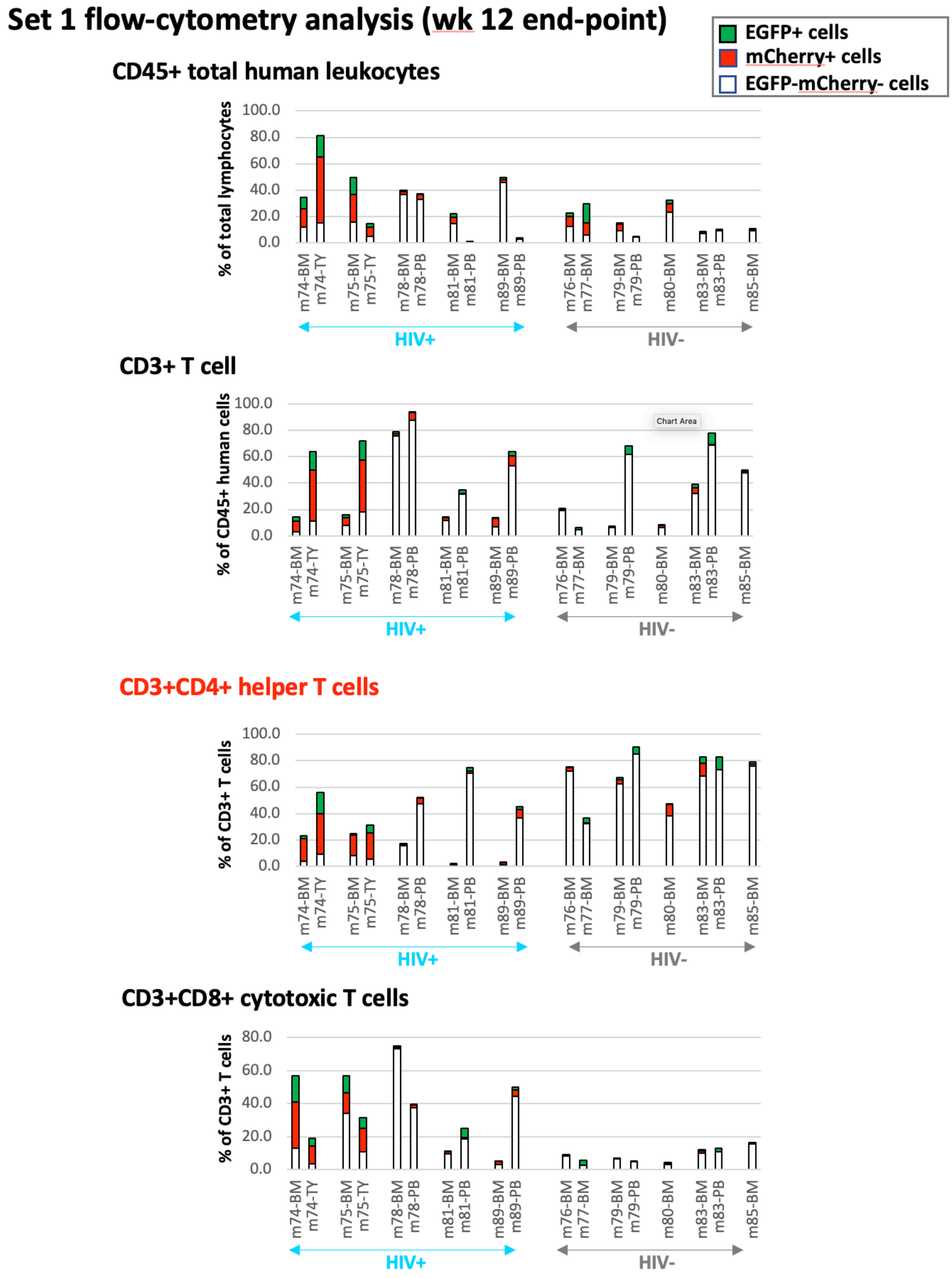

Supplementary Figure S1 (d)

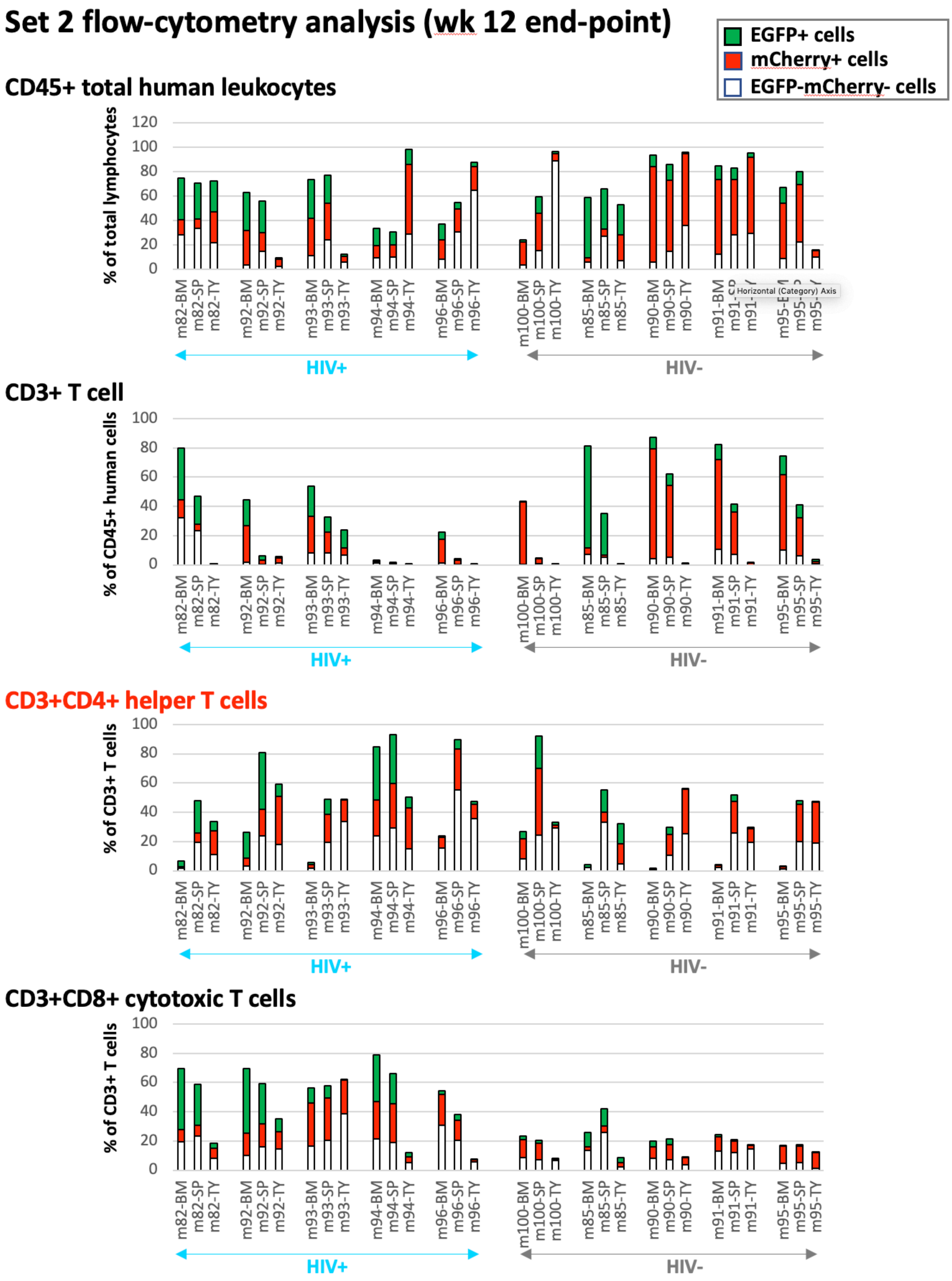

Supplementary Figure S1 (e)

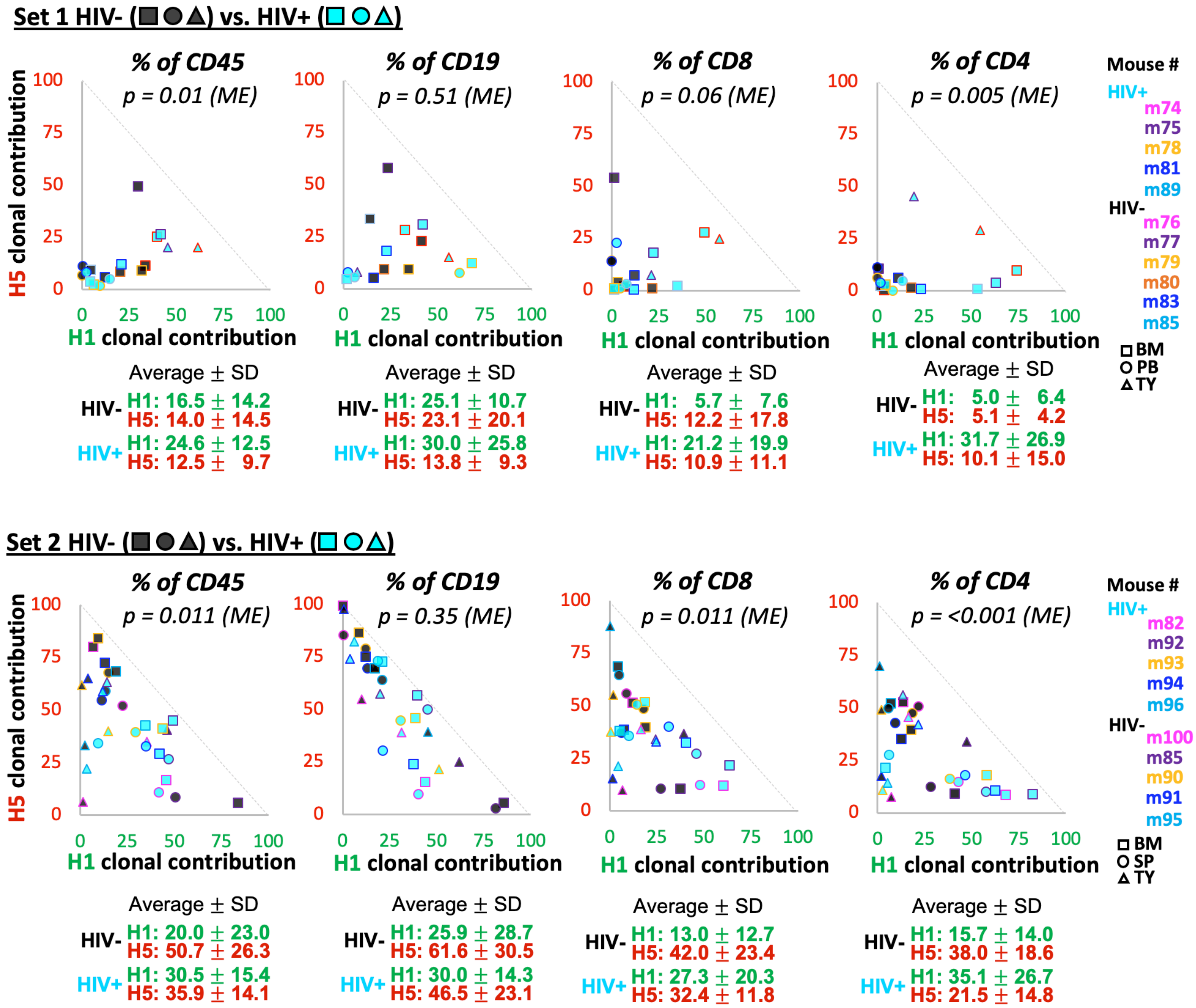

Supplementary Figure S1: Flow cytometry analysis (a-b) Flow cytometry analysis at 6, 8, and 10 weeks post BLT surgery. The percentage of EGFP+, mCherry+, and EGFP-mCherry- cell populations in various human blood cell lineages, including CD45+ leukocytes, CD3+ T cells, CD3+CD4+ T helper cells, CD3+CD8+ cytotoxic T cells and CD19+ B cells in HIV-1 infected (HIV+) and mock-infected (HIV-) mice are shown at 6 weeks (wk 6), 8 weeks (wk 8), and 10 weeks (wk 10) post-BLT/HSPC transplant for Set 1(**a**) and Set 2 (**b**). **(c-d) Flow cytometry analysis results at the week 12 end-point.** Bone marrow (BM), peripheral blood (PB), and thymic organelle (TY) repopulating cells are shown for Set 1 (**c**) and BM, TY, and spleen (SP) repopulating cells are shown for Set 2 (**d**). **(e) Paired H1-EGFP and H5-mCherry markings in BM (squares), TY (triangles), PB (circles in Set 1), and SP (circles in Set 2) in HIV-1 infected (cyan shapes) and mock-infected mouse samples (black shapes)**. Percentage of H5-mCherry (y-axis) and percentage of H1-EGFP (x-axis) are shown for different human cell lineages, including total human leukocytes (CD45), B cells (CD19), CD3+CD8+ cytotoxic T cells (CD8), and CD3+CD4+ T helper cells (CD4). The line colors of squares, triangles, and circles indicate mouse IDs. *p* values were calculated by mixed effects gamma regressions (see Supplemental Data). Average ± standard deviation (SD) markings are shown at the bottom of each plot.

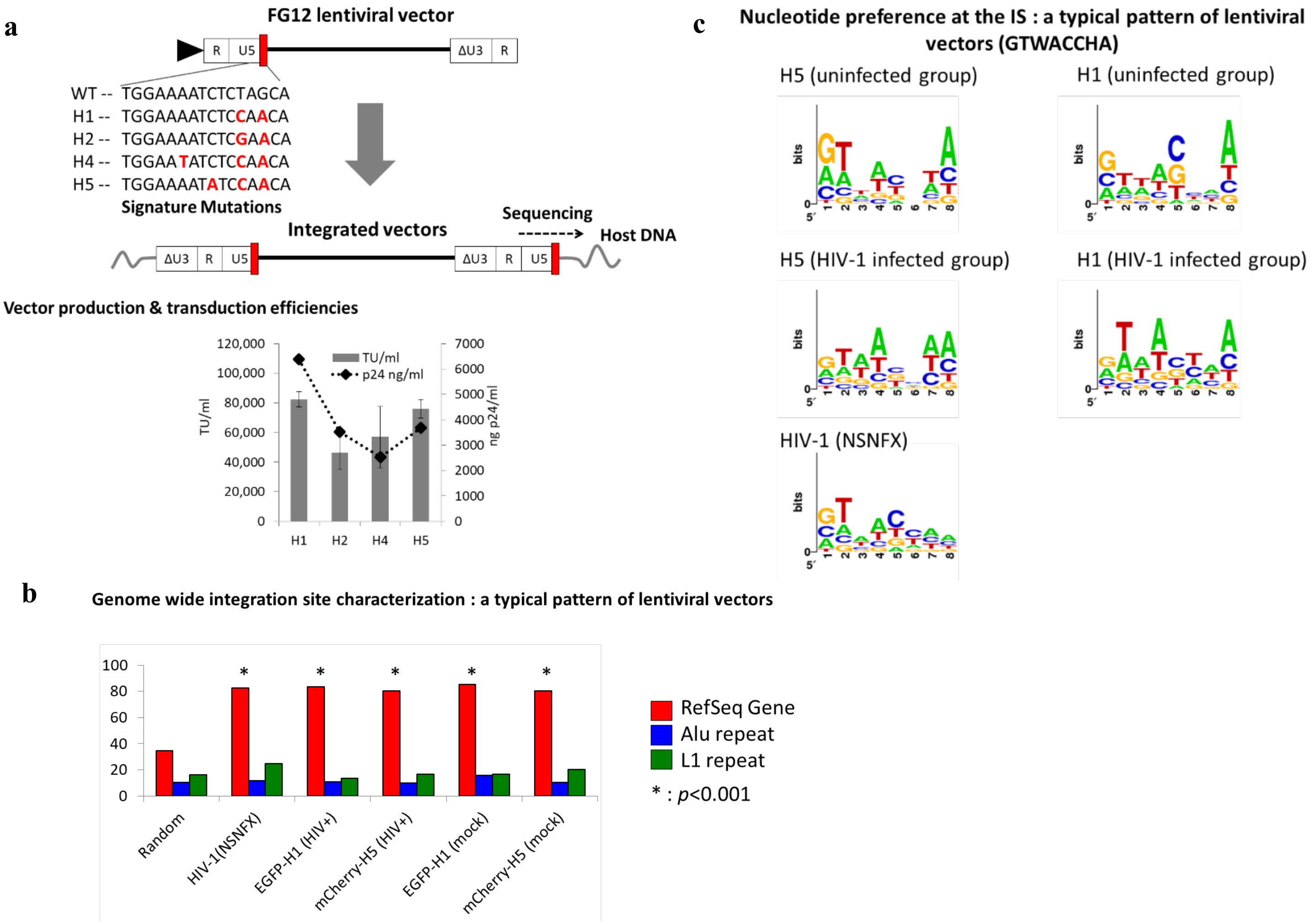

Supplementary Figure S2: Testing lentiviral vectors with different LTR index (LTRi) mutations (a) LTRi vectors. Top figures show different LTR index sequences that were tested for transduction efficiency and vector titer. Bottom figure shows data for vector titer and transduction efficiency. H1, H2, H4, and H5 vectors were generated by introducing two or three nucleotide mutations at the 3' end of the U5 LTR of FG12 lentiviral vector. These LTRi mutations will appear at the junction of the vector and host DNA after vector integration into the host genome and will thus serve as a marker with which to distinguish vector types during vector integration site sequence analysis. All H1, H2, H4, and H5 lentivirus vectors were produced by calcium phosphate-mediated transient transfection of 293T cells as described previously by Ringpis et al. (21), and Shimizu et al. (20) and Morizono et al. (55). Virus stocks were titrated by infecting 10^5 293T cells with various dilutions of the concentrated virus stock and followed by flow cytometry analysis of EGFP expression on 3 days post-infection. (**b**) **Integration site distribution patterns.** Genome wide characterization of integration site for R5 HIV-1_{NSNFX} and the two lentiviral vectors, H1-EGFP-Dual-shRNA and H5-mCherry (control) in HIV infected (HIV+) and un-infected (HIV-1) mice. The relative integration site frequencies within the RefSeq gene, Alu, and L1 repeats are shown in comparison to those of random integration sites, indicating predominant integration sites within RefSeq gene regions. **(c)**. **Nucleotide preferences at the IS target sites**. First to eighth nucleotide positions of the integration site are shown for R5 trophic HIV-1_{NSNFX}, H1-EGFP-Dual-shRNA, and FG12_H5_mCherry vectors in HIV infected (HIV+) and un-infected (HIV-1) BLT mice. A preference to the GTWACCHA motif, a typical pattern of lentiviral vectors is shown for all IS datasets.

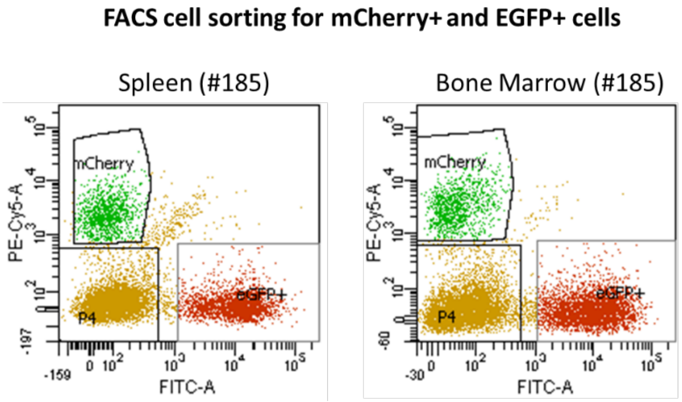

Supplementary figure S3: Flow sorting for mCherry+ and GFP+ cells for conventional vector integration site analysis. mCherry+ and GFP+ spleen and bone marrow cells from BLT mice were sorted using FACSAria II cell sorter (BD Biosciences).

(a)

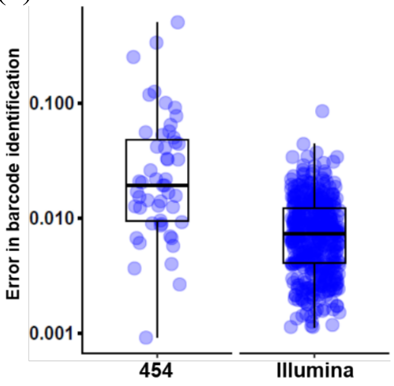

(b)

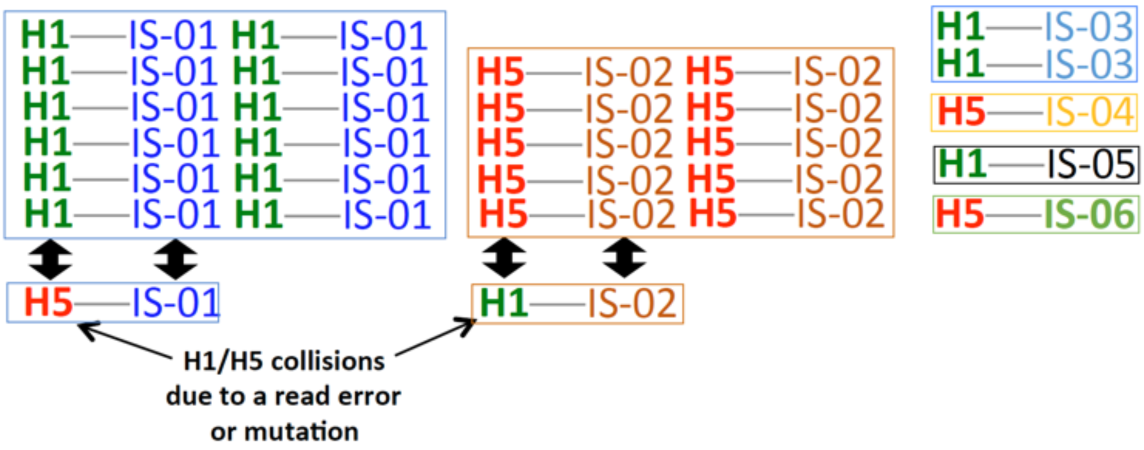

Supplementary Figure S4: LTR index read errors (a) LTR index read errors in 454-pyrosequencing and Illumina sequencing data. Boxplots show ranges of the nucleotide substitution error rates for LTR index sequences that led to LTR index collisions in Set 1 454-pyrosequencing (454) and Set 2 Illumina sequencing data (Illumina). Index read error rates were about 1.92% and 0.73% for Set 1 and Set 2, respectively (**b**) **Identification and correction of index read errors.** In most cases, index read errors can be readily identified by comparing the sequence counts of the two collision groups that the share same IS (e.g. see IS-01 and IS-02 groups above). LTR index collisions were effectively resolved with our 10X collision correction criteria (see Methods for details), leaving only 0.7% of Set 1 IS events and no Set 2 IS events unresolved. The unresolved IS clones were removed from the final analysis. Low-copy IS clones that did not show collision events had approximately 1.92% and 0.73% uncertainty (or less) in their LTR index identities due to the intrinsic read errors of the sequencing platforms.

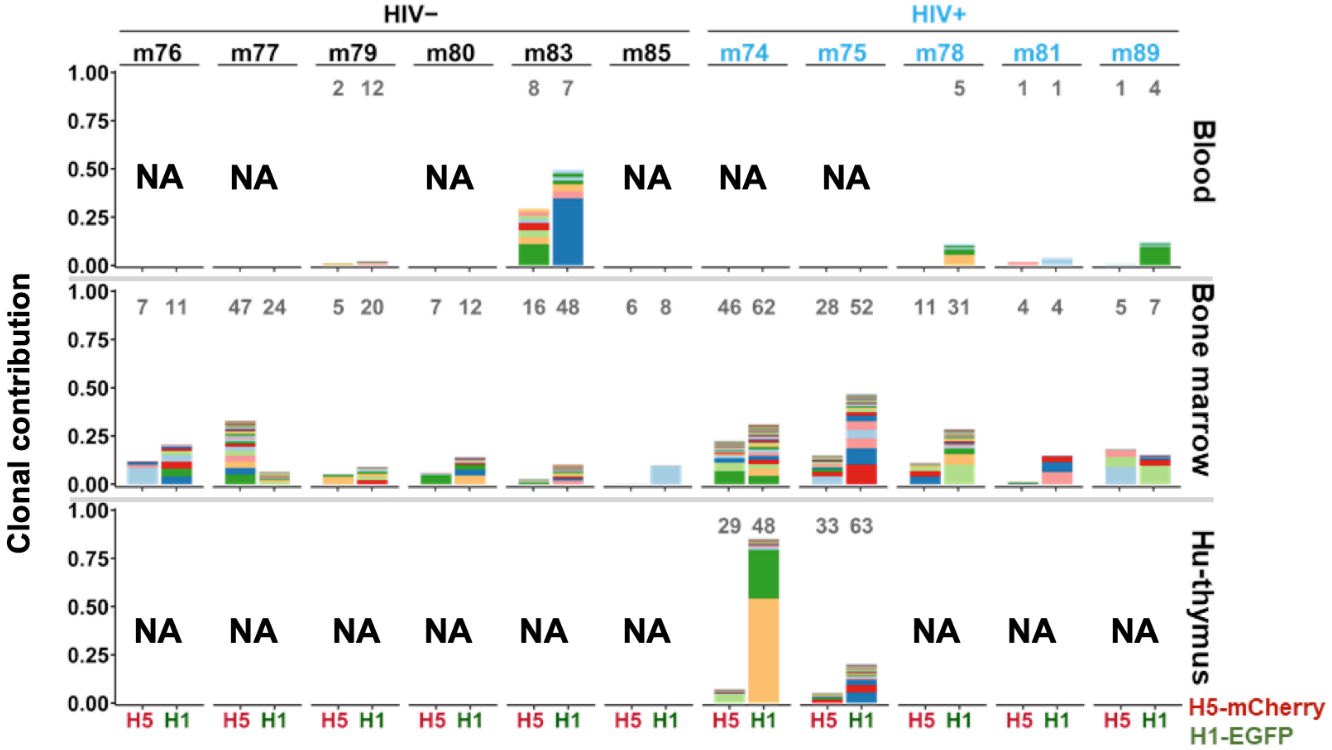

Supplementary figure S5: Stacked area plots for Set 1 IS clones. H1-EGFP-dual-shRNA (H1) and H5-mCherry (H5) clones at the end point (week 12) are shown for different organs, including the peripheral blood, bone marrow, spleen, and human thymic organelle (Hu-thymus), of HIV- and HIV+ mice in Set 1 experiments. H1 and H5 IS frequencies were normalized with total vector markings (fractions of EGFP+ and mCherry+ cells in CD45+ cells) and are shown as clonal contribution (y-axis). Each color band appearing in a stack represents an individual IS clone. The total number of IS clones (grey numbers) and mouse IDs (black or cyan texts) are shown at the top of the chart. NA means not available.

(a)

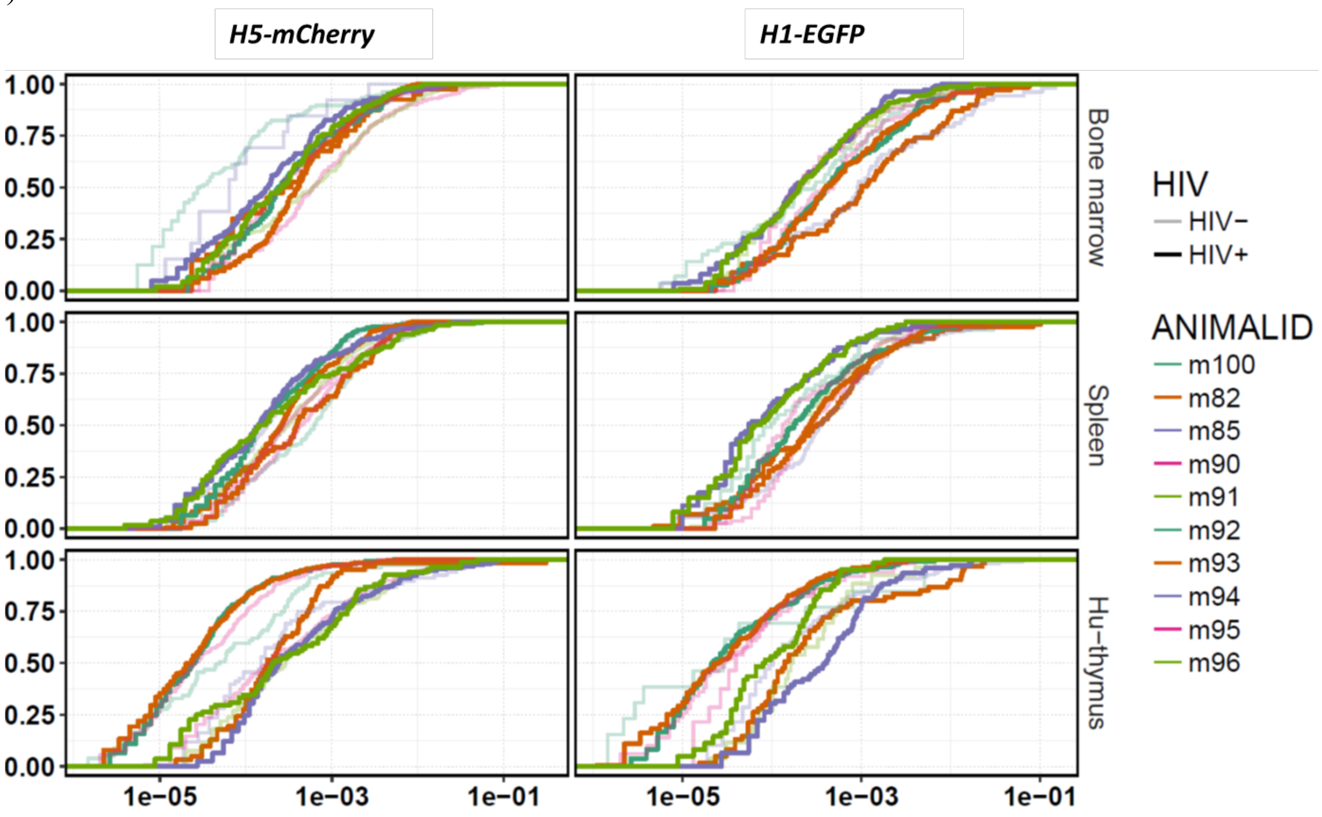

(b)

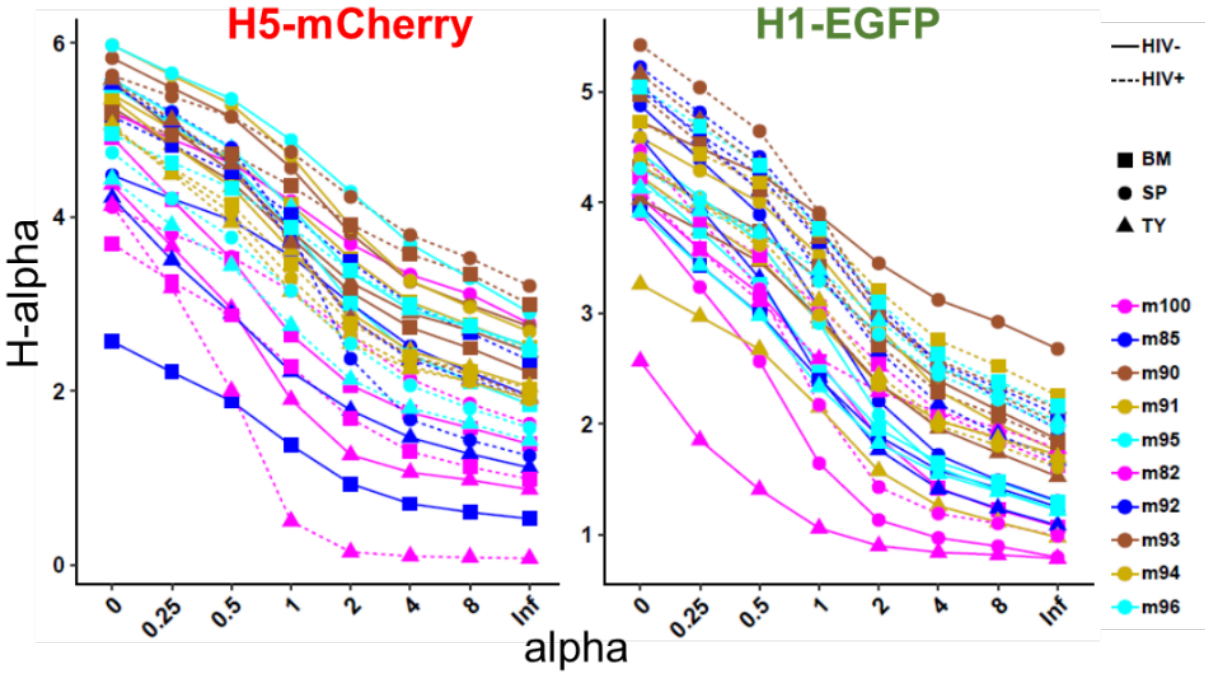

Supplementary figure S6: Clonal diversity analysis. (**a**) **Line plots showing cumulative clonal size distribution of H5-mCherry and H1-EGFP IS clones in different organs/tissues for each animal in Set 2 experiment**. Different color line show data for different mice. Faded lines are for HIV- mice and dark line are for HIV+ mice. Cumulative curves were created with the "ecdf" function in R software as described previously by Goyal et al.(47) (**b**) **Rényi's diversity profiles**. For the diversity analysis we used

Rényi's diversity/entropy of order α for $\alpha > 0$ and $\alpha \neq 1$, defined as follows [see Renyi, et al. 1961 (48) and Tóthmérész, et al. 1995 (59)].

$$H_\alpha = \frac{1}{1-\alpha}\log\left(\sum p_i^\alpha\right),$$

where p_i is the proportional abundance of the ith IS clone for $i = 1, \dots, n$. Rényi's diversity profile is obtained by evaluating values of H_α for α in the range of 0 to ∞. The α can also be considered as a weighing parameter such that the influence of any dominant IS increases with increasing α. The proportional abundance is calculated as $p_i = s_{i,X}/S_{\mathrm{X}}$, where $s_{i,X}$ is the sequence count of ith IS clone of type X and S_X is the sum of sequence counts from all type X IS clones. The Rényi's diversity values H_α are plotted as function of α. If all IS clones contribute equally, i.e. $p_i = \frac{1}{n}$ for all $i = 1, \dots, n$, then H_α for all values of α will be equal and the profile will be horizontal. A set of IS clones expanding at different rates shows decreasing H_α values as α increases, generating a sloped diversity profile. For $\alpha = 0$, $H_0 = \log(n)$ and the antilogarithm of this value equates to the richness or number of unique IS. We created the Renyi's diversity plots using the R package BiodiversityR (*https://cran.r-project.org/web/packages/BiodiversityR/index.html*)[see Kindt and Coe, 2005 (60)]. For this we used raw sequence counts with and without distinguishing between H5-mCherry IS and H1-EGFP IS. H5 (upper panel) and H1 (lower panel) IS clonal size distributions are shown for bone marrow (BM; squares), spleen (SP; circles), and thymus (TY; triangles) of individual HIV- (continuous line) and HIV+ mice (dotted line) in Set 2.

a

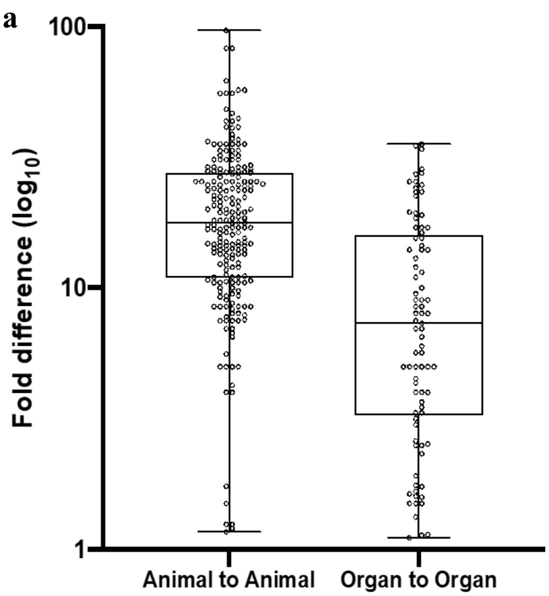

	Mann-Whitney test	
1	Table Analyzed	Data 4
2		
3	Column B	Organ to Organ
4	vs.	vs.
5	Column A	Animal to Animal
6		
7	**Mann Whitney test**	
8	P value	<0.0001
9	Exact or approximate P value?	Exact
10	P value summary	****
11	Significantly different (P < 0.05)?	Yes
12	One- or two-tailed P value?	Two-tailed
13	Sum of ranks in column A,B	36609 , 7942
14	Mann-Whitney U	4287
15		
16	**Difference between medians**	
17	Median of column A	17.75, n=213
18	Median of column B	7.330, n=85
19	Difference: Actual	-10.42
20	Difference: Hodges-Lehmann	-9.214
21		
22		
23		

b

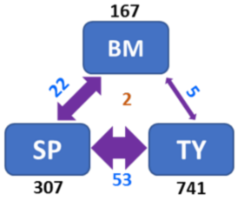

1,

	SP	TY
Common with BM	22	5
Not common with BM	285	736

P<0.001 – reject

2,

	SP	BM
Common with TY	53	5
Not common with TY	254	162

P<0.001 – reject

3,

	TY	BM
Common with SP	53	22
Not common with SP	688	145

P<0.0165 – reject

Supplementary Figure S7: It is unlikely that the organ-to-organ IS distribution patterns are the results of simple random contamination or sequencing errors. (**a**) **The fold difference of IS sequence counts (y-axis) among organ-to-organ crossover events and animal-to-animal crossover events.** The fold difference were determined by comparing sequence counts between the highest IS frequency event and all the others sharing the same IS. As expected, the sequence count differences for animal-to-animal crossovers were significantly higher than those of organ-to-organ crossovers (p = <0.0001, Mann Whitney test), indicating the organ-to-organ IS crossover events are less likely the results of simple random contamination. Random IS collisions resulting from contamination or sequencing errors (e.g. animal-to-animal) will likely show higher sequence count differences than those of physiological crossovers among organs due to cellular migrations. Our data showed that, of a total of 1,178 HIV-1 IS recovered, 84 showed crossover in different animals (10 animals) and 82 showed crossover in different organs (3 organs). (**b**) **Organ-to-organ IS crossover events are not randomly distributed**. In the context of the 3 organs in this study, we tested the hypothesis that the likelihood of having common IS in a given organ pair would be same for the other two pairs. This was broken down to three null hypotheses to test: (1) The likelihood of having common IS in both bone marrow (BM) and spleen (SP) is same as that in BM and Thymic organelle (Thy). (2) The likelihood of having common IS in Thy and SP is same as that in Thy and BM. (3) The likelihood of having common IS in SP and Thy is same as that in SP and BM. If any of these are rejected, the null hypothesis is rejected. Three chi-square tests at p=0.05 level were performed. All three null hypotheses were rejected. Bonferroni's method was used to adjust for multiple comparisons (reject if p<0.017).

Supplementary Figure S8 (a)

(a-i-Set 1)

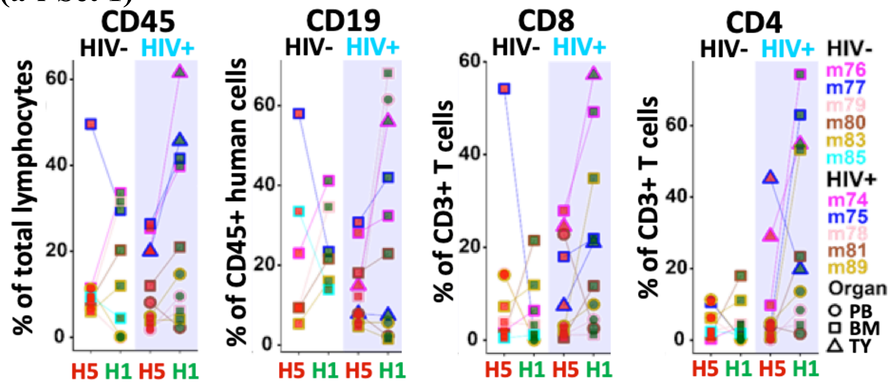

(a-i-Set 2)

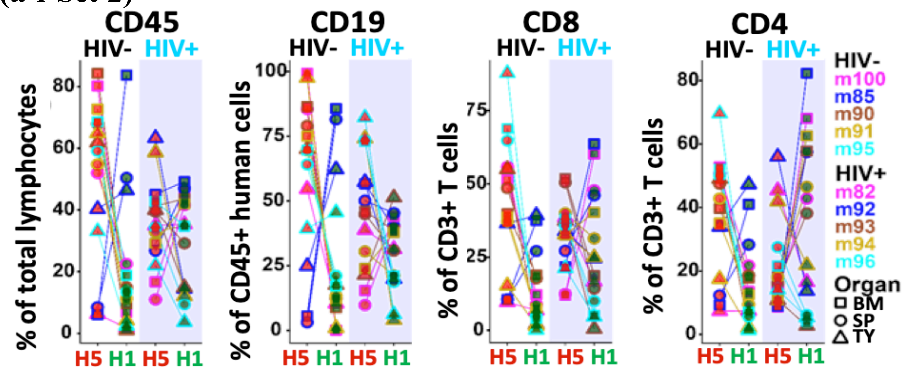

(a-ii-Set 1)

lineages	Variables	Ratio (95% CI)	P value
CD45	H1/H5 in HIV- mice	0.9 (0.4,1.7)	0.66
CD45	H5 in HIV+ / H5 in HIV-	0.7 (0.4,1.4)	0.37
CD45	(H5 in HIV-):BM/SP	3.3 (2.0,5.6)	<0.001
CD45	(H5 in HIV-):TY/SP	5.7 (2.5,13.0)	<0.001
CD45	*H1/H5 in HIV+ vs. H1/H5 in HIV-)	*2.2 (0.9,5.3)	*0.10
-	*the H1/H5 ratio is 2.2 times greater (p = 0.10) in HIV+ than that in HIV-	-	-
CD3.	H1/H5 in HIV- mice	0.5 (0.2,1.6)	0.24
CD3.	H5 in HIV+/ H5 in HIV-	0.8 (0.3,2.2)	0.71
CD3.	(H5 in HIV-):BM/SP	2.4 (1.0,5.6)	0.043
CD3.	(H5 in HIV-):TY/SP	5.5 (1.6,19.1)	0.007
CD3.	*H1/H5 in HIV+ vs. H1/H5 in HIV-)	*5.5 (1.4,21.7)	*0.015
-	*the H1/H5 ratio is 5.5 times greater (p = 0.015) in HIV+ than that in HIV-	-	-
CD19	H1/H5 in HIV- mice	1.3 (0.6,2.7)	0.47
CD19	H5 in HIV+/ H5 in HIV-	0.8 (0.3,2.2)	0.69
CD19	(H5 in HIV-):BM/SP	1.6 (0.8,3.2)	0.20
CD19	(H5 in HIV-):TY/SP	0.9 (0.3,2.7)	0.88
CD19	*H1/H5 in HIV+ vs. H1/H5 in HIV-)	*1.4 (0.5,3.4)	*0.51
-	*the H1/H5 ratio is 1.4 times greater (p = 0.51) in HIV+ than that in HIV-	-	-
CD8	H1/H5 in HIV- mice	0.3 (0.1,1.2)	0.09
CD8	H5 in HIV+/ H5 in HIV-	0.8 (0.3,2.3)	0.67
CD8	(H5 in HIV-):BM/SP	2.2 (0.8,5.7)	0.11
CD8	(H5 in HIV-):TY/SP	3.5 (0.8,14.4)	0.08
CD8	*H1/H5 in HIV+ vs. H1/H5 in HIV-)	*4.4 (1.0,20.6)	*0.06
-	*the H1/H5 ratio is 4.4 times greater (p = 0.06) in HIV+ than that in HIV-	-	-
CD4	H1/H5 in HIV- mice	0.7 (0.2,2.1)	0.51
CD4	H5 in HIV+/ H5 in HIV-	0.7 (0.3,2.1)	0.57
CD4	(H5 in HIV-):BM/SP	2.1 (0.8,5.0)	0.11
CD4	(H5 in HIV-):TY/SP	8.3 (2.3,30.6)	0.001
CD4	*H1/H5 in HIV+ vs. H1/H5 in HIV-)	*7.6 (1.8,31.6)	*0.005
	*the H1/H5 ratio is 7.6 times greater (p = 0.005) in HIV+ than that in HIV-		

(a-ii-Set 2)

Cell	Variables	Ratio (95% CI)	p.value
CD45	H1/H5 in HIV- mice	0.3 (0.2,0.5)	<0.001
CD45	H5 in HIV+/ H5 in HIV-	0.7 (0.4,1.2)	0.21
CD45	*H1/H5 in HIV+ vs. H1/H5 in HIV-)	*2.3 (1.2,4.3)	*0.011
-	*the H1/H5 ratio is 2.3 times greater (*p* = 0.011) in HIV+ mice than that in HIV-	-	-
CD3	H1/H5 in HIV- mice	0.3 (0.2,0.6)	<0.001
CD3	H5 in HIV+/ H5 in HIV-	0.8 (0.5,1.5)	0.53
CD3	*H1/H5 in HIV+ vs. H1/H5 in HIV-)	*2.1 (1.0,4.6)	*0.053
-	*the H1/H5 ratio is 2.1 times greater (*p* = 0.053) in HIV+ mice than that in HIV-	-	-
CD19	H1/H5 in HIV- mice	0.4 (0.2,0.8)	0.006
CD19	H5 in HIV+/ H5 in HIV-	0.8 (0.4,1.4)	0.38
CD19	*H1/H5 in HIV+ vs. H1/H5 in HIV-)	*1.5 (0.6,3.6)	*0.35
-	*the H1/H5 ratio is 1.5 times greater (*p* = 0.35) in HIV+ mice than that in HIV-	-	-
CD8	H1/H5 in HIV- mice	0.3 (0.1,0.5)	<0.001
CD8	H5 in HIV+/ H5 in HIV-	0.8 (0.4,1.4)	0.40
CD8	*H1/H5 in HIV+ vs. H1/H5 in HIV-)	*2.8 (1.3,6.2)	*0.011
-	*the H1/H5 ratio is 2.8 times greater (*p* = 0.011) in HIV+ mice than that in HIV-	-	-
CD4	H1/H5 in HIV- mice	0.4 (0.2,0.7)	0.001
CD4	H5 in HIV+/ H5 in HIV-	0.6 (0.3,1.1)	0.08
CD4	*H1/H5 in HIV+ vs. H1/H5 in HIV-)	*3.9 (1.8,8.4)	*<0.001
	*the H1/H5 ratio is 3.9 times greater (*p* = <0.001) in HIV+ mice than that in HIV-		

(a-iii)

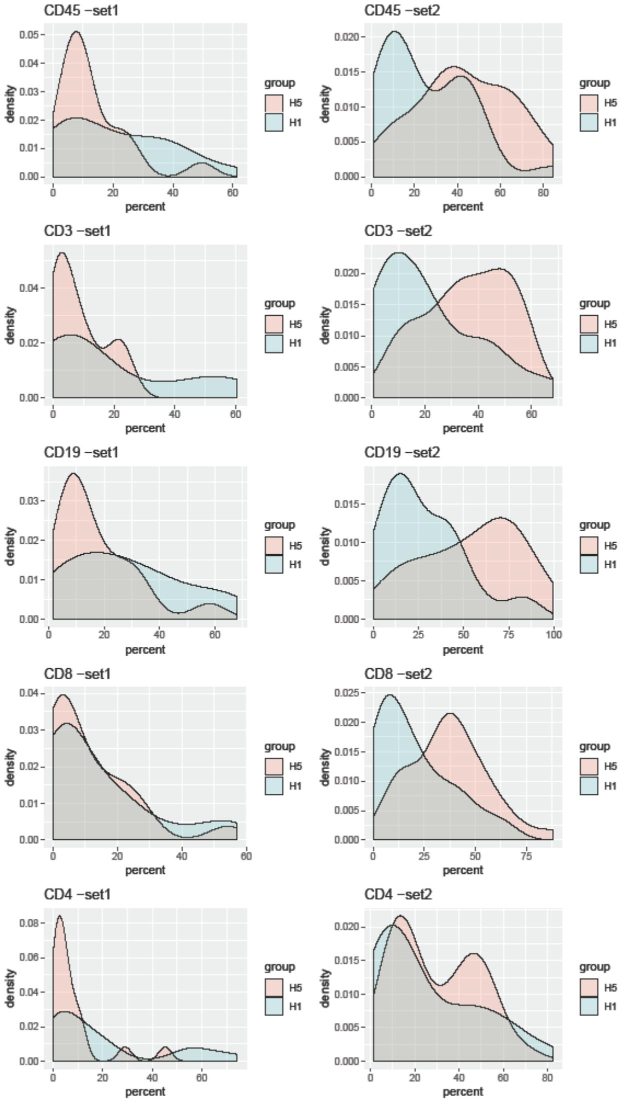

Supplementary Figure S8 (b)

(b-i)

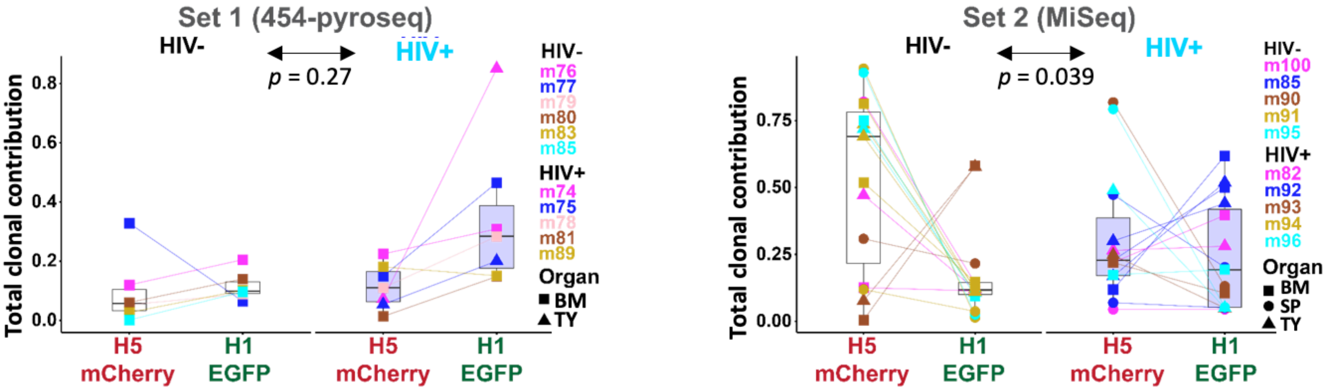

(b-ii)

Set	Variables	Ratio (95% CI)	p.value
1	H1/H5 in HIV- mice	1.5 (0.5,4.6)	0.52
	H5 in HIV+/ H5 in HIV-	1.3 (0.5,3.7)	0.61
	TissueHu-thymus	0.9 (0.3,2.6)	0.84
	*H1/H5 in HIV+ vs. H1/H5 in HIV-	*2.1 (0.6,7.8)	*0.27
-	*the H1/H5 ratio is 2.1 times greater (*p* = 0.27) in HIV+ than that in HIV-	-	-
2	H1/H5 in HIV- mice	0.3 (0.2,0.6)	<0.001
	H5 in HIV+/ H5 in HIV-	0.6 (0.3,1.1)	0.08
	TissueHu-thymus	0.8 (0.5,1.4)	0.46
	TissueSpleen	1.0 (0.6,1.7)	0.95
	*H1/H5 in HIV+ vs. H1/H5 in HIV-	*2.4 (1.0,5.7)	*0.039
	*the H1/H5 ratio is 2.4 times greater (*p* = 0.039) in HIV+ than that in HIV-		

(b-iii-)

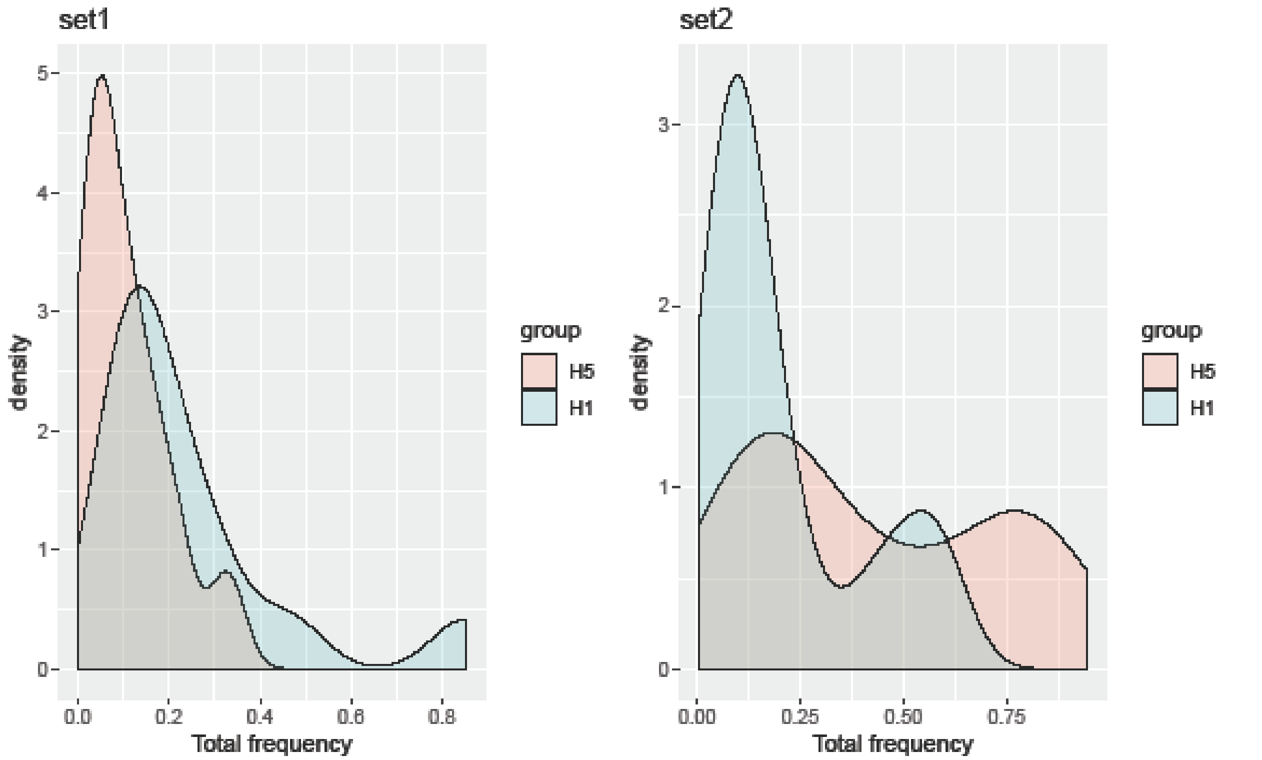

Supplementary Figure S8 (c)

(c-i) **(Set 1)** **(Set 2)**

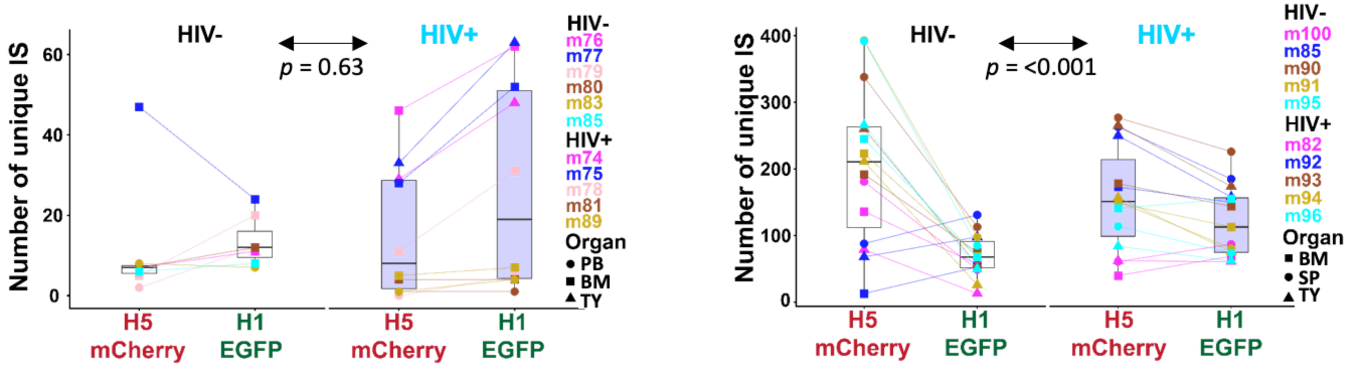

(c-ii)

Set	Variables	Ratio (95% CI)	p.value	Variables	Ratio (95% CI)	p.value
1	H1/H5 in HIV-[1]	1.7 (0.9,3.0)[1]	0.09[1]	H1/H5 in HIV+[3]	2.0 (1.2,3.2)[3]	0.004[3]
	H5 in HIV+/H5 in HIV-[2]	0.9 (0.3,2.9)[3]	0.85[3]	H1 in HIV+/H1 in HIV-[4]	1.1 (0.3,3.5)[4]	0.91[4]
	TissueBM	3.9 (2.3,6.5)	<0.001			
	TissueThy	4.1 (1.7,9.7)	0.002			
	H1/H5 ratios in HIV+ vs. HIV-[5]	1.2 (0.6,2.5)	0.63			
	-	-	-	-	-	-
2	H1/H5 in HIV-[1]	0.4 (0.3,0.5)[1]	<0.001[1]	H1/H5 in HIV+[3]	0.8 (0.6,1.05)[2]	0.11[2]
	H5 in HIV+/H5 in HIV-[2]	0.7 (0.4,1.4)[2]	0.36[3]	H1 in HIV+/H1 in HIV-[4]	1.7 (1.1,2.6)[4]	0.010[4]
	TissueBM	1.3 (1.0,1.7)	0.037			
	TissueThy	0.9 (0.7,1.2)	0.52			
	H1/H5 ratios in HIV+ vs. HIV-[5]	2.2 (1.5,3.4)	<0.001			

[1]The H1 IS number for HIV- mice vs H5 IS number in HIV- mice. This is estimated from a model with H5 and HIV- being the reference levels.

[2]From the same model and same reference levels, we also estimated the H5 IS number in HIV+ vs H5 IS number HIV- mice.

[3]The H1 IS number in HIV+ mice vs. H5 IS number in HIV+ mice. This is estimated from the same model but with H5 and HIV+ being the reference levels.

[4]From the same model and same reference levels, we also estimated the H1 IS number in HIV+ vs H1 IS number HIV-. This is estimated from the same model but with H1 and HIV- being the reference levels.

[5]The H1/H5 ratio is 1.2 times (p = 0.63) greater in HIV+ mice compared to in HIV- mice in Set 1 and 2.2 times (p = <0.001) greater in Set 2.

(c-iii)

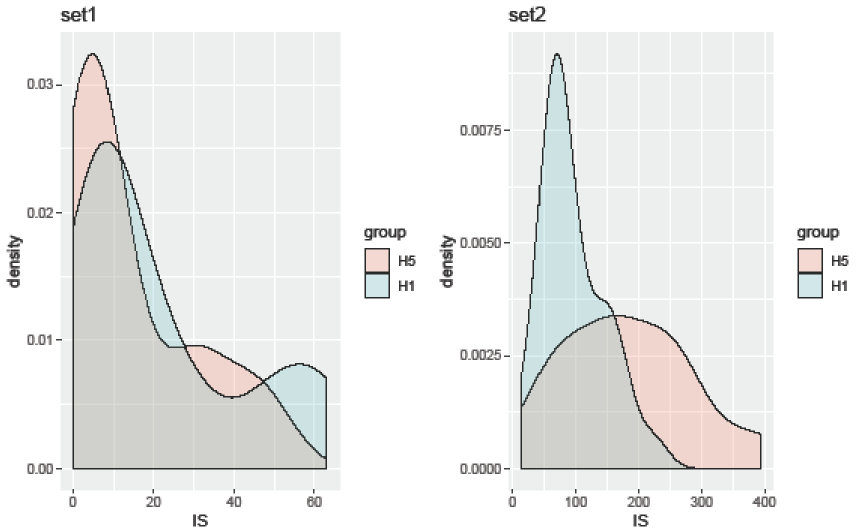

Supplementary Figure S8: Mixed Effects Gamma Regression Models. (**a**) **Evaluating the impact of HIV-1 infection on H1-EGFP+ and H5-mCherry+ cells**. Paired data for % vector marking of EGFP (H1) and mCherry (H5) within total human leukocytes (CD45), B cells (CD19), CD3+CD8+ cytotoxic T cells (CD8), and CD3+CD4+ T helper cells (CD4), in different organs of uninfected (HIV-) and HIV infected (HIV+) mice at Week 12 are shown for Set 1 (**a-xi**) and Set 2 (**a-xii**). We used mixed effects gamma regressions to compare the ratios of % EGFP(H1) and % mCherry(H5) between HIV+ and HIV- mice adjusting for tissue type. The models included an interaction term between H1(vs. H5) and HIV status to test whether the effect of H1(vs. H5) differed by HIV status. Correlation among measurements from the same animal was assumed to be unstructured. For cell%, when the outcome measure was 0 a small number (0.1) was used instead to meet the range requirement of gamma regression. Ratios, 95% confidence intervals (CIs) and p-values were reported from the models. Statistical significance was assessed at the 0.05 level and analyses were implemented in R v.3.4.4 (https://www.R-project.org/)(70). Mixed effects gamma regression comparing the % ratio of H1 and H5 and HIV+/- is in the form of *"% cells ~ (H1 vs H5)*(HIV infected +/-) + tissue type|mouse ID*". A separate model was run for each of the blood lineages (CD45+, CD3+, CD19+, CD3+CD8+, CD3+CD4+) of Set 1 (**a-yi**) and Set 2 (**a-yii**). The plots of data outcome are shown for Set 1 (**a-zi**) and Set 2 (**a-zii**). (**b**) **Evaluating the impact of HIV-1 infection on the total contributions of H1 and H5 clones**. *Paired H5 and H1 total IS clonal contribution (linked by a colored line) comparing HIV- and HIV+ mouse samples (**b-i**). Set 1 data are shown on the left and Set 2 on the right.* Model is in the form of "*Total frequency ~ (H1 vs H5)*(HIV infected +/-) + tissue type|mouse ID*" (**b-ii**). The plots of data outcome are shown (**b-iii**). (**c**) **Evaluating the impact of HIV-1 infection on the total number of H1 and H5 clones.** Paired H5 and H1 IS numbers in organ samples, each linked by a colored line for Set 1 (left) and Set 2 (right) *(c-i).* Model is in the form of "Number of unique IS ~ (H1 vs H5)*(HIV infected +/-) + tissue type|mouse ID" (c-ii). The plots of data outcome are shown (c-**iii**).

Supplementary Table S1. HIV-1 viral load data for Set 1 and Set 2 mice.

	Mouse #	before BLT (copies/ml)	after BLT (end point) (copies/ml)
Set 1 HIV+	m74 (BS3)	491,000	5,210,000
	m75 (BS3)	503,000	3,360,000
	m78 (BS3)	684,000	2,130,000
	m81 (BS3)	169,000	715,000
	m89 (BS3)	704,000	339,000
Set 2 HIV+	M82 (BS24)	340,000	1,700,000
	M92 (BS24)	28,000,000	33,000,000
	M93 (BS24)	30,000,000	10,000,000
	M94 (BS24)	39,000,000	39,000,000
	M96 (BS24)	22,000,000	24,000,000

Supplementary Table S2. Summary of IS sequencing

	Infection	Animal ID#	Tissue	gDNA (µg)^	Total IS seqs*	Unique IS$	EGFP IS$	mCherry IS$	HIV-1 IS$
**Set 1**	HIV+	m74	Bone marrow	1	1,294	117	62	46	9
			Hu-thymus	1	4,568	81	48	29	4
			Total	-	5,862	186	102	71	13
		m75	Bone marrow	1	2,028	93	52	28	13
			Hu-thymus	1	1,419	133	63	33	38
			Total	-	3,447	197	96	52	49
		m78	Bone marrow	1	570	79	31	11	37
			Blood	0.67	12	5	5	0	0
			Total	-	582	81	33	11	37
		m81	Bone marrow	1	228	10	4	4	2
			Blood	0.45	8	4	1	1	2
			Total	-	236	13	5	4	4
		m89	Bone marrow	1	110	12	7	5	0
			Blood	0.36	30	5	4	1	0
			Total	-	140	16	10	6	0
	HIV-	m79	Bone marrow	1	868	25	20	5	0
			Blood	1	452	14	12	2	0
			Total	-	1,320	28	23	5	0
		m83	Bone marrow	1	1,184	66	48	16	2
			Blood	0.87	44	16	7	8	1
			Total	-	1,228	76	50	23	3
		m76	Bone marrow	1	632	20	11	7	2
		m77	Bone marrow	1	1,767	72	24	47	1
		m80	Bone marrow	1	1,552	21	12	7	2
		m85	Bone marrow	1	2,236	14	8	6	0
**Set 2**		Animal ID#	Tissue	gDNA (µg)^	Total IS seqs*	Unique IS$	EGFP IS$	mCherry IS$	HIV-1 IS$
	HIV-	m100	Bone marrow	2	76,246	193	57	136	0
			Spleen	2	49,434	230	49	181	0
			Hu-thymus	2	105,719	92	13	79	0
			Total	-	231,399	276	69	207	0
		m85	Bone marrow	2	90,293	69	53	13	3
			Spleen	2	94,426	219	131	88	0
			Hu-thymus	2	48,544	166	98	68	0
			Total	-	233,263	276	160	113	3
		m90	Bone marrow	2	61,202	268	75	192	1
			Spleen	2	60,442	452	113	338	0
			Hu-thymus	2	91,033	316	56	260	0
			Total	-	212,677	542	141	400	1

		m91	Bone marrow	2	68,314	294	69	223	2
			Spleen	2	89,013	492	98	393	1
			Hu-thymus	2	64,742	237	26	211	0
			Total	-	222,069	632	130	499	3
		m95	Bone marrow	2	31,029	313	68	245	0
			Spleen	2	57,123	477	85	392	0
			Hu-thymus	2	55,219	316	50	266	0
			Total	-	143,371	655	131	524	0
	HIV+	m82	Bone marrow	2	39,253	126	69	40	17
			Spleen	2	77,661	182	87	61	34
			Hu-thymus	2	65,422	413	61	62	290
			Total	-	182,336	532	119	83	330
		m92	Bone marrow	2	55,692	374	150	173	51
			Spleen	2	46,540	524	185	263	76
			Hu-thymus	2	54,714	555	158	250	147
			Total	-	156,946	880	254	369	257
		m93	Bone marrow	2	66,483	342	144	178	20
			Spleen	2	45,755	629	226	277	126
			Hu-thymus	0.58	61,190	543	174	265	104
			Total	-	173,428	871	283	362	226
		m94	Bone marrow	2	59,100	319	113	149	57
			Spleen	2	41,285	272	81	151	40
			Hu-thymus	2	50,504	362	76	157	129
			Total	-	150,889	613	161	237	215
		m96	Bone marrow	2	62,971	318	155	141	22
			Spleen	2	60,439	219	74	114	31
			Hu-thymus	2	50,727	217	62	84	71
			Total	-	174,137	450	176	165	109

# identification numbers for Set 1 animals (Animal ID)
^the amount of genomic DNA used in the analysis [gDNA (μg)]
**the total number of integration site sequences (Total IS seqs)*
$ the total number of unique integration sites (IS)
